# Identification of a novel sesquiterpene biosynthetic machinery involved in astellolide biosynthesis

**DOI:** 10.1038/srep32865

**Published:** 2016-09-15

**Authors:** Yasutomo Shinohara, Shunji Takahashi, Hiroyuki Osada, Yasuji Koyama

**Affiliations:** 1 Noda Institute for Scientific Research, 399 Noda, Noda, Chiba 278-0037, Japan; 2 Chemical Biology Research Group, RIKEN CSRS, 2-1 Hirosawa, Wako, Saitama 351-0198, Japan

## Abstract

Esterified drimane-type sesquiterpene lactones such as astellolides display various biological activities and are widely produced by plants and fungi. Given their low homology to known sesquiterpene cyclases, the genes responsible for their biosynthesis have not been uncovered yet. Here, we identified the astellolide gene cluster from *Aspergillus oryzae* and discovered a novel sesquiterpene biosynthetic machinery consisting of AstC, AstI, and AstK. All these enzymes are annotated as haloacid dehalogenase-like hydrolases, whereas AstC also contains a DxDTT motif conserved in class II diterpene cyclases. Based on enzyme reaction analyses, we found that AstC catalysed the protonation-initiated cyclisation of farnesyl pyrophosphate into drimanyl pyrophosphate. This was successively dephosphorylated by AstI and AstK to produce drim-8-ene-11-ol. Moreover, we also identified and characterised a unique non-ribosomal peptide synthetase, AstA, responsible for esterifying aryl acids to drimane-type sesquiterpene lactones. In this study, we highlight a new biosynthetic route for producing sesquiterpene and its esterified derivative. Our findings shed light on the identification of novel sesquiterpenes via genome mining.

Secondary metabolites (SMs) produced by filamentous fungi have distinct biological activities; some have beneficial effects on human health (e.g., penicillin and lovastatin), whereas others pose a risk to food safety (e.g., aflatoxins and ochratoxins)^1^,^2^. Recent advances in genome sequencing and SM biosynthetic gene cluster prediction have indicated that filamentous fungi have more clusters and greater SM-producing ability than previously anticipated^3^,^4^.

*Aspergillus oryzae* is a filamentous fungus widely used in the production of traditional Japanese fermented foods. It is thought that as a result of domestication, it lacks the ability to produce mycotoxins, such as aflatoxin^5^,^6^, aflatrem^7^, and cyclopiazonic acid^8^,^9^. The genetic causes underlying the inability to produce mycotoxins have been well characterised. Recent genomic analyses have revealed a large number of SM gene clusters in the *A. oryzae* genome. A better understanding of *A. oryzae* SMs and the genes responsible for their production has important implications not only for human health and food safety, but also for the discovery of new drugs.

The *cclA* gene is involved in regulating the production of several SMs in *A. nidulans*^10^ and *A. fumigatus*^11^. We recently demonstrated that disruption of *cclA* in *A. oryzae* also altered the SM production profile, increasing the amount of astellolides, aryl acid esterified drimane-type sesquiterpene lactones, formerly known as parasiticolides^12^. Drimane-type sesquiterpene esters, including astellolides, are widely distributed in plants^13^,^14^ and fungi^15^,^16^. Some of them have shown antimicrobial, anti-inflammatory, and anti-tumour activities^12^,^13^,^14^,^15^,^16^. However, the corresponding biosynthetic pathways and genes have not been identified yet. In this study, we used a disruption mutant to identify the astellolide biosynthetic gene cluster in *A. oryzae*. Furthermore, we used purified recombinant enzymes to functionally characterise a novel pathway involved in astellolide biosynthesis; this includes a sesquiterpene cyclase, dephosphorylases, and an ester-forming enzyme.

## Results

### Identification of the gene cluster responsible for astellolide biosynthesis

**Figure 1:**
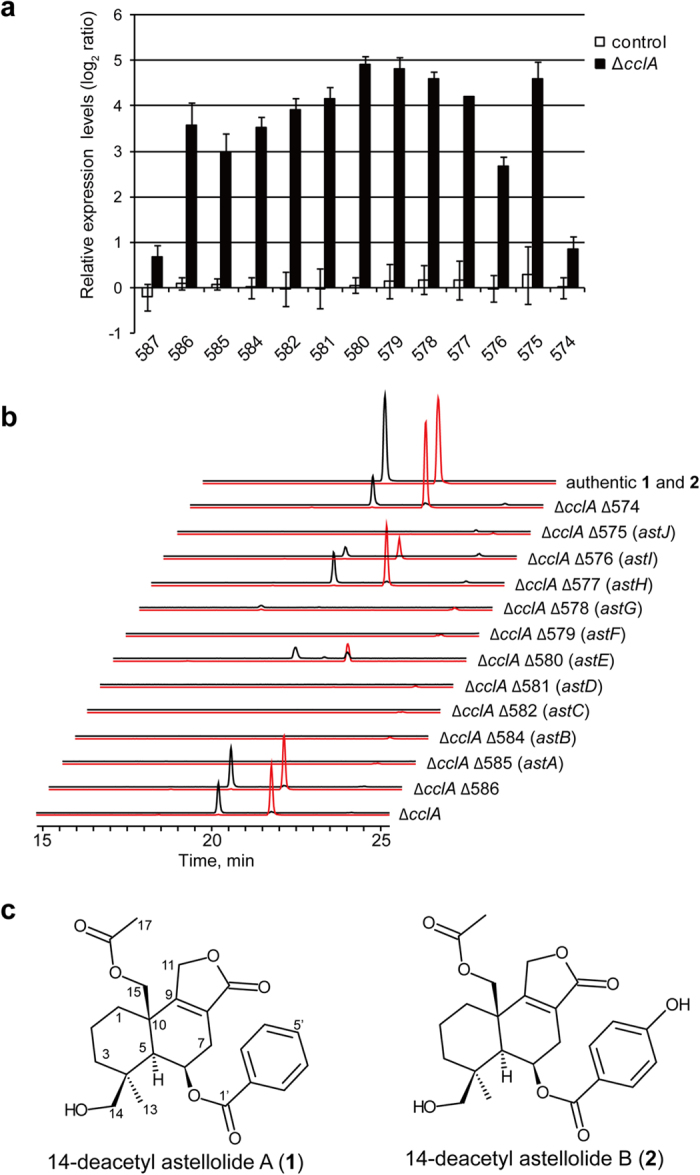
Expression analysis and genetic validation of the putative astellolide biosynthetic gene cluster. (**a**) Quantitative real-time PCR analysis of the putative genes involved in astellolide biosynthesis. X-axis labels indicate the abbreviated gene ID (e.g., 585 represents the abbreviated version of the gene ID AO090026000585). Histone 2B was used as an endogenous reference gene. Data are presented as mean ± SD (n = 3). (**b**) Extracted ion chromatograms of **1** (*m*/*z* 429 [M + H]^+^, red) and **2** (*m*/*z* 445 [M + H]^+^, black) in culture extracts from gene disruption strains, and authentic **1** and **2**^12^. Twelve genes were disrupted in the Δ*cclA* background; each resultant disruption strain was named Δ*cclA*Δ*X*, where “*X*” represents the abbreviated gene ID. (**c**) Chemical structures of **1** and **2**.

Secondary metabolite gene clusters have been previously predicted using various bioinformatics tools such as Secondary Metabolite Unknown Regions Finder (SMURF)^3^. In this study, we first investigated the gene expression profile of a *cclA* disruption strain (Δ*cclA*) using custom-made gene expression arrays. We found that SMURF-predicted cluster39 was clearly upregulated relative to the control RkuptrP2-1ΔAF/P strain. Quantitative real-time PCR (qRT-PCR) confirmed that the genes spanning the region between AO090026000586 and AO090026000575 of this cluster were overexpressed in the Δ*cclA* strain (Fig. 1a). To determine whether this cluster was involved in the production of astellolides, we disrupted each gene in a Δ*cclA* background. The metabolite profiles of the disrupted strains were analysed by liquid chromatography/electrospray ionisation mass spectrometry (LC/ESI-MS). We found that seven out of 12 genes examined lacked 14-deacetyl astellolide A (**1**) and B (**2**) (Fig. 1b,c). The production of **1** and **2** was not affected by the disruption of AO090026000574. Moreover, although AO090026000586 was upregulated in the Δ*cclA* strain (Fig. 1a), its disruption did not affect the production of **1** and **2** (Fig. 1b). Therefore, we concluded that cluster39 was involved in the production of astellolides. We also speculated that the region between AO090026000585 and AO090026000575 was an astellolide biosynthetic gene cluster and named the corresponding genes *astA* to *astJ* (Table 1).

### Characterisation of sesquiterpene cyclase

**Figure 2:**
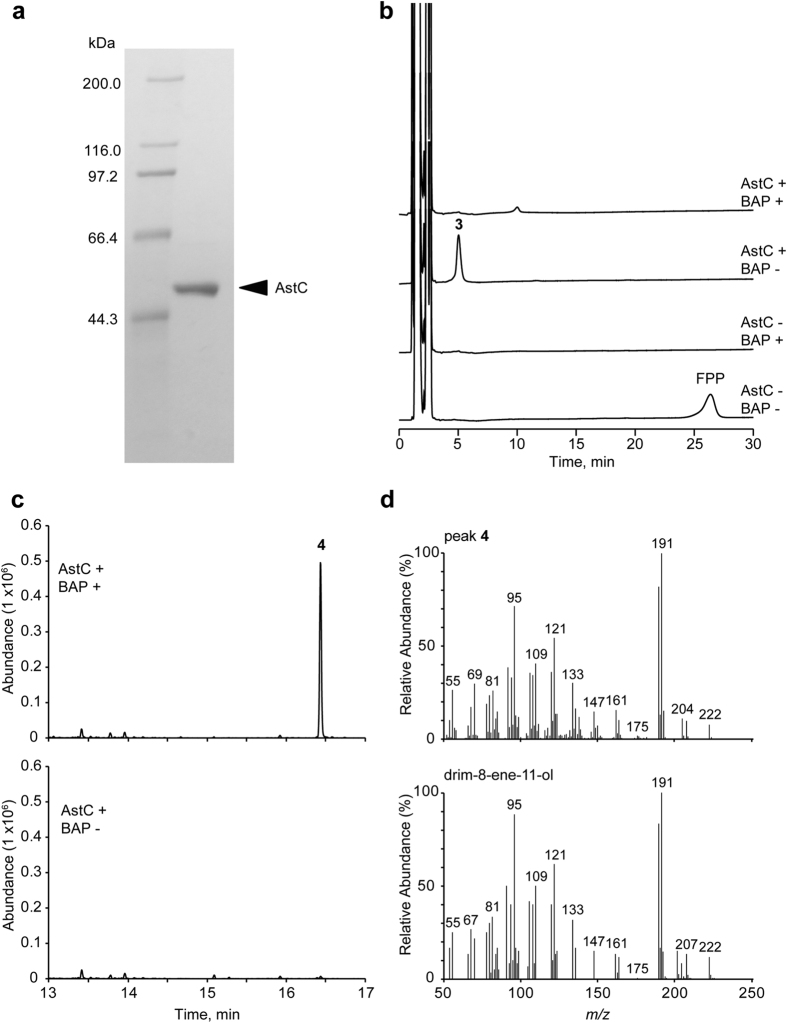
Functional characterisation of AstC. (**a**) SDS-PAGE analysis of purified AstC. (**b**) HPLC analysis (UV 210 nm) of the reaction product of AstC with FPP. After incubation of AstC with FPP for 1 h, alkaline phosphatase (BAP) was added and further incubated for 1 h. (**c**) GC-MS analysis of the reaction product of AstC with or without alkaline phosphatase treatment. (**d**) MS spectra of the product peak (**4**) (upper panel) and reference MS spectra of drim-8-ene-11-ol from the Wiley 9th edition NIST11 (W9N11) mass spectral library (lower panel).

The drimane-type sesquiterpene backbone found in astellolides suggested the presence of a terpene cyclase in the cluster, even though BLAST searches did not return any putative hit (Table 1). However, a search for conserved motifs revealed that the amino acid sequence of AstC contained a DxDTT motif. This is a variation of the DxDD motif, which is conserved in class II diterpene cyclase^17^,^18^ (Supplementary Fig. 1). To examine whether AstC had sesquiterpene cyclase activity, we purified it (Fig. 2a) and performed the AstC reaction in the presence of farnesyl pyrophosphate (FPP). High performance liquid chromatography (HPLC) revealed that product **3** peaked at 5.0 min, coinciding with disappearance of the FPP peak (Fig. 2b, second top panel). Interestingly, the peak of product **3** was lost after alkaline phosphatase treatment, suggesting the presence of a pyrophosphate group (Fig. 2b, top panel). We treated the AstC reaction mixture with or without alkaline phosphatase; ethyl acetate extracts were analysed by gas chromatography (GC)-MS. As expected, a single peak at 16.4 min was detected in the extracts of the alkaline phosphatase-treated reaction (Fig. 2c). By comparing the product’s MS spectrum with the W9N11 MS library, we speculated that the compound may be drim-8-ene-11-ol (**4**) (Fig. 2d). To confirm the presence of a pyrophosphate group in **3**, we applied reverse-phase HPLC and purified **3** from a large-scale reaction mixture without alkaline phosphatase treatment. The molecular formula of **3** was established as C_15_H_27_O_7_P_2_ by high resolution electrospray ionization (HRESI)-MS (*m*/*z* 381.1223, [M - H]^−^). ^1^H and ^13^C nuclear magnetic resonance (NMR) analyses confirmed that the structure of the sesquiterpene portion of **3** was identical to **4** (Supplementary Table 1). The presence of a pyrophosphate group in **3** was confirmed by ^31^P NMR (202 MHz): δ −6.9 (d, J = 22 Hz) and δ −10.7 (d, J = 22 Hz), using phosphoric acid as an external standard at 0 ppm. Additionally, the ^13^C NMR spectral data showed that the signals of C-9 (δ 140.1) and C-11 (δ 64.3) were split into doublets with coupling constants of 9.5 and 4.2 Hz, respectively, by coupling with ^31^P. Based on these findings, we hypothesised that AstC had sesquiterpene cyclase activity and generated drimanyl pyrophosphate (**3**) via protonation-initiated cyclisation.

### Characterisation of sesquiterpene phosphatases

**Figure 3:**
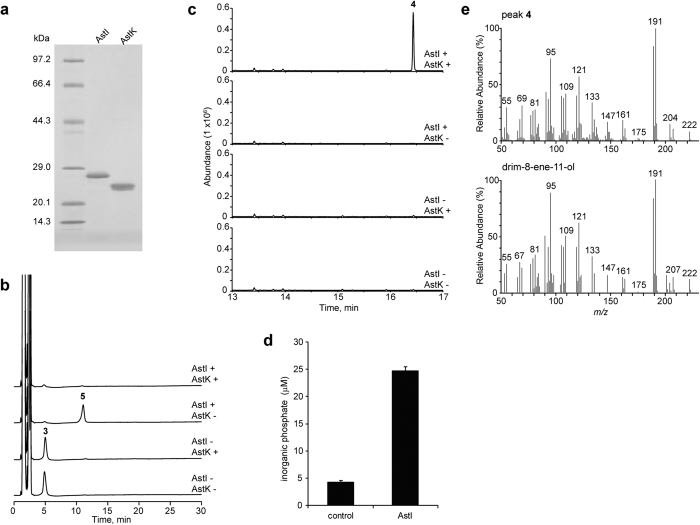
Functional characterisation of AstI and AstK. (**a**) SDS-PAGE analysis of purified AstI and AstK. (**b**) HPLC analysis (UV 210 nm) of the reaction products of AstI, AstK, or both with **3**. (**c**) GC-MS analysis of the reaction products of AstI, AstK, or both with **3**. (**d**) Amount of inorganic phosphate released by incubating AstI with **3** for 30 min. Data are presented as mean ± SD (n = 3). (**e**) MS spectra of the product peak (**4**) (upper panel) and reference MS spectra of drim-8-ene-11-ol from the W9N11 mass spectral library (lower panel).

The AstC reaction strongly suggested that the biosynthesis of astellolides required a specific and efficient depyrophosphorylation enzyme. We first focused on AstI, a member of the haloacid dehalogenase (HAD)-like hydrolase superfamily, some of whose members are known to act as phosphatases^19^. We purified AstI to homogeneity (Fig. 3a) and tested its depyrophosphorylation activity upon incubation with **3**. HPLC analysis revealed that product **5** peaked at 11.1 min along with a decrease in **3** (Fig. 3b, second chromatogram from the top). Given that the reaction product corresponding to **4** was not detected by GC-MS (Fig. 3c, second top panel), we speculated that **5** was drimanyl monophosphate. To elucidate the function of AstI, inorganic phosphate (Pi) was measured using the malachite green assay. As expected, incubation of AstI with the AstC reaction mixture resulted in a significant increase in the Pi concentration (Fig. 3d). This finding suggested the presence of another enzyme catalysing the dephosphorylation of **5**.

Recently, the revised annotation of the *A. oryzae* RIB40 genome was released by the National Institute of Technology and Evaluation (NITE) (http://www.bio.nite.go.jp/dogan/project/view/AO2). Accordingly, two additional genes (AORIB40_NS.05916 and AORIB40_NS.05917) adjacent to AO090026000575 (*astJ*) were predicted (Table 1 and Supplementary Fig. 2). Interestingly, AORIB40_NS.05916 (named *astK*) was also expected to be a member of the HAD-like hydrolase superfamily. To identify the missing drimanyl monophosphate dephosphorylase, we purified AstK (Fig. 3a) and incubated it together with AstI in the presence of the AstC reaction mixture. HPLC analysis showed no phosphorylated product (Fig. 3b, top chromatogram), however GC-MS analysis revealed a peak corresponding to **4** (Fig. 3c, top panel, and 3e). The results strongly suggested that AstI and AstK catalysed the successive dephosphorylation of **3** into **4** via **5**.

### Identification of genes involved in ester bond formation and acetylation

**Figure 4:**
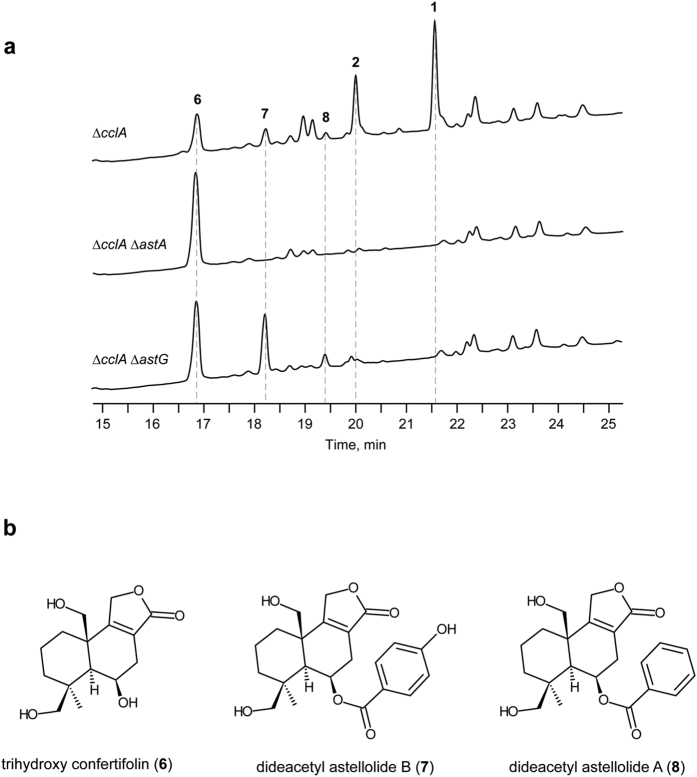
Metabolite profiles of culture extracts from the *astA* or *astG* disruption strains. (**a**) HPLC profiles of the culture extracts of the *cclA* disruption (Δ*cclA*), the *cclA* and *astA* double-disruption (Δ*cclA* Δ*astA*), and the *cclA* and *astG* double-disruption (Δ*cclA* Δ*astG*) strains. UV detection was performed at 220 nm. (**b**) Chemical structures of **6**, **7**, and **8** (see Supplementary Table 2; Supplementary Fig. 3).

When compared to the Δ*cclA* strain, LC/ESI-MS analysis of extracts from the Δ*cclA* Δ*astA* double disruption strain showed the absence of **1** and **2**, and the presence of an enhanced peak at 16.7 min corresponding to compound **6** (Fig. 4a). The Δ*cclA* Δ*astG* strain also lacked **1** and **2**. This mutant accumulated two compounds (**7** at 18.2 min and **8** at 19.4 min) along with **6** (Fig. 4a). To determine their structures, we isolated **6** from a large-scale culture of the Δ*cclA* Δ*astA* strain, and **7** and **8** from that of the Δ*cclA* Δ*astG* strain. HRESI-MS analysis of **6** showed a [M + H]^+^ ion at *m*/*z* 283.1563, corresponding to the molecular formula C_15_H_22_O_5_. From its ^1^H and ^13^C NMR spectra, **6** was identified as a novel trihydroxylated derivative of confertifolin, which was previously isolated from plants such as *Drimys* species^20^, and named trihydroxy confertifolin (Fig. 4b; see also Supplementary Table 2 and Supplementary Fig. 3). On the contrary, HRESI-MS analysis of **7** and **8** showed a [M + H]^+^ ion at *m/z* 403.1764 and 387.1811, respectively, which is consistent with the corresponding deacetylated derivatives **2** and **1**. As expected, NMR analysis identified **7** and **8** as C15-deacetylated derivatives of **2** and **1**, respectively (Fig. 4b; see also Supplementary Table 2 and Supplementary Fig. 3). Product **8** was recently isolated from *A. oryzae* RIB40^15^. These data suggest that AstA catalyses ester bond formation between **6** and benzoic acid (BA) or 4-hydroxy benzoic acid (4HBA) to form **7** and **8**. They also indicate, that AstG promotes *O*-acetylation at position C-15 of **7** and **8** to form **2** and **1**, respectively.

### Characterisation of an ester-forming enzyme

**Figure 5:**
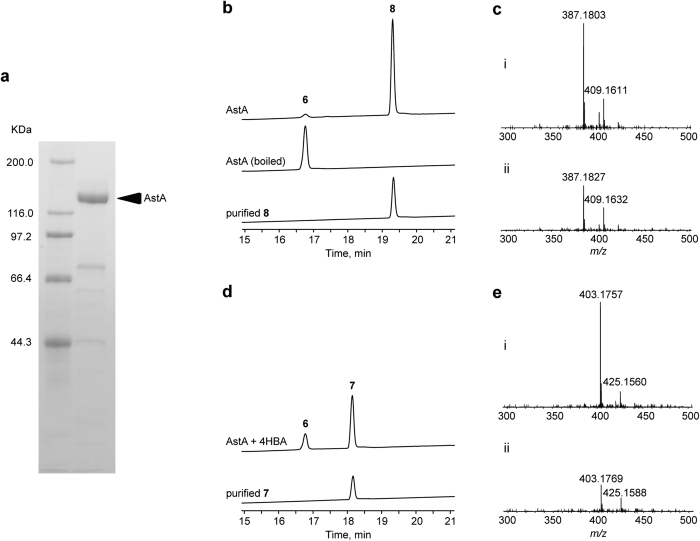
*In vitro* reconstitution of the esterification of aryl acids by AstA. (**a**) SDS-PAGE analysis of purified AstA. (**b**) HPLC analysis (UV 220 nm) of the reaction products of AstA in the presence of BA and **6**, and purified **8**. Boiled AstA was used as a negative control. (**c**) MS spectra of the reaction product of AstA with BA and **6** (i), and purified **8** (ii). (**d**) HPLC analysis (UV 220 nm) of the reaction product of AstA in the presence of 4HBA and **6**, and purified **7**. (**e**) MS spectra of the reaction product of AstA with 4HBA and **6** (i), and purified **7** (ii).

Domain analysis against the Pfam database indicated that AstA encoded a non-ribosomal peptide synthetase (NRPS) enzyme containing adenylation (A), thiolation (T), and condensation (C) domains. However, the accumulation of **6** in the Δ*cclA* Δ*astA* strain suggested that AstA might catalyse ester bond formation. To test this possibility, we heterologously expressed AstA in *Escherichia coli* BL21 (Fig. 5a). Purified AstA was incubated with **6** and BA. LC/ESI-MS analysis of the reaction product revealed that AstA was able to convert **6** into a product with *m/z* 387.2 [M + H]^+^ and 409.2 [M + Na]^+^, which was consistent with purified **8** (Fig. 5b,c). Considering that the Δ*cclA* Δ*astA* strain failed to produce both **1** and **2** (Fig. 4a), we also performed an AstA assay using 4HBA as a substrate. LC/ESI-MS analysis revealed that AstA converted **6** into a product with *m*/*z* 403.2 [M + H]^+^ and 425.2 [M + Na]^+^, which was consistent with purified **7** (Fig. 5d,e). Moreover, we performed steady-state kinetic analysis using the pyrophosphate release assay. Prior to the reaction, we confirmed that modification of the T-domain by 4′-phosphopantetheinyl transferase (Sfp) was essential (Supplementary Fig. 4). The observed *K*_*m*_ and *k*_cat_ values were 4.9 μM and 1.33 min^−1^ for BA, and 3.8 μM and 0.98 min^−1^ for 4HBA, respectively, suggesting that the A-domain of AstA had a similar substrate preference for BA and 4HBA.

### Analysis of putative P450, dehydrogenase, and transporter gene disruptants

The astellolide biosynthetic gene cluster contains four genes (*astB*, *astD, astF*, and *astJ*) predicted to encode cytochrome P450 (Table 1). The disruption of *astB*, *astF*, and *astJ* genes resulted in the loss of **1**, **2**, and **6** (Fig. 1b, Supplementary Fig. 5a). LC/ESI-MS analysis showed that the disruption strains accumulated novel compounds (*m*/*z* 267.2) with different retention times (Δ*cclA* Δ*astB* 18.7 min, Δ*cclA* Δ*astF* 17.2 min, and Δ*cclA* Δ*astJ* 17.9 min, respectively) (Supplementary Fig. 5b). The ion with *m*/*z* 267.2 corresponded to the proton adduct of a dihydroxylated derivative of confertifolin. Given the high homology to P450, AstB, AstF, and AstJ may contribute to the hydroxylation of confertifolin at loci C6, C14, or C15 to form **6**.

In contrast, the disruption of another P450 gene, *astD*, resulted in the loss of biosynthetic intermediates (Supplementary Figs. 5a–c). One possible explanation is that AstD may participate in the biosynthesis of confertifolin and the volatile intermediate **4**, both of which were lost during the extraction procedure. *astE* disruption strain showed a significant reduction of **1** and **2** (Fig. 1b), suggesting that the loss of AstE, a predicted short-chain dehydrogenase, might be partially substituted by unknown enzymes. Based on metabolite analyses of all gene disruptions in the cluster, we could not obtain clear intermediates involved in the conversion of **4** into confertifolin. However, we speculate that the uncharacterised enzymes, AstE and AstD, might be involved in lactone formation. The *astH* disruption strain continued to produce **1** and **2** (Fig. 1b), suggesting that AstH, a predicted major facilitator superfamily transporter, did not participate in the translocation of astellolides.

## Discussion

There are two types of terpene cyclases based on the production mechanism of the initial carbocation: (1) an “ionisation-initiated’’ (type-A/class I) mechanism, which generates a carbocation by the release of a pyrophosphate group via the conserved DDxxD/E motif; and (2) a “protonation-initiated” (type-B/class II) mechanism, which generates a carbocation by protonating a double-bond via the conserved DxDD (DxDTT) motif. Although sesquiterpene cyclases typically catalyse cyclisation via the ionisation-initiated mechanism^21^,^22^, drimane-type sesquiterpene cyclisation is explained by the protonation-initiated mechanism^16^. Recently, Kwon *et al*. reported the cloning and characterisation of a plant drimenol cyclase containing a typical ionisation-initiated motif^23^; however, the underlying catalytic mechanism was not elucidated in their study. Based on detailed analyses of the role of AstC in astellolide biosynthesis, here we provide the first evidence of drimane-type sesquiterpene cyclisation via a protonation-initiated mechanism.

Plant and fungal terpene cyclases present very low overall sequence homology, except for conserved catalytic domains, such as DDxxD/E and DxDD (DxDTT) motifs^24^,^25^. For example, AstC shares only 15% overall sequence identity to the diterpene cyclase Rv3377c from *Mycobacterium tuberculosis* H37^17^. Rv3377c contains a DxDTT motif found in the protonation-initiated type cyclase. Moreover, AstC shares a very low sequence identity with fungal bifunctional diterpene cyclases^26^,^27^,^28^,^29^, which also harbour the catalytic domain of the protonation-initiated type. Interestingly, a BLAST search indicated the existence of AstC orthologs containing the DxDTT motif in a number of fungal species, including Basidiomycetes and Ascomycetes. Sequence alignment of AstC and orthologous proteins revealed the existence of the QW motif (Qxx(D/G)G(G/S)W), a known conserved domain in terpene cyclases^18^,^30^. Although DDxxE, the motif conserved in ionisation-initiated type terpene cyclase, was found in some of the AstC orthologs, it showed a substitution of the second Asp for Asn in AstC (Supplementary Fig. 1). Knowing that the second Asp is important for catalytic activity^31^,^32^, we expected the loss of ionisation-initiated cyclisation activity by AstC.

**Figure 6:**
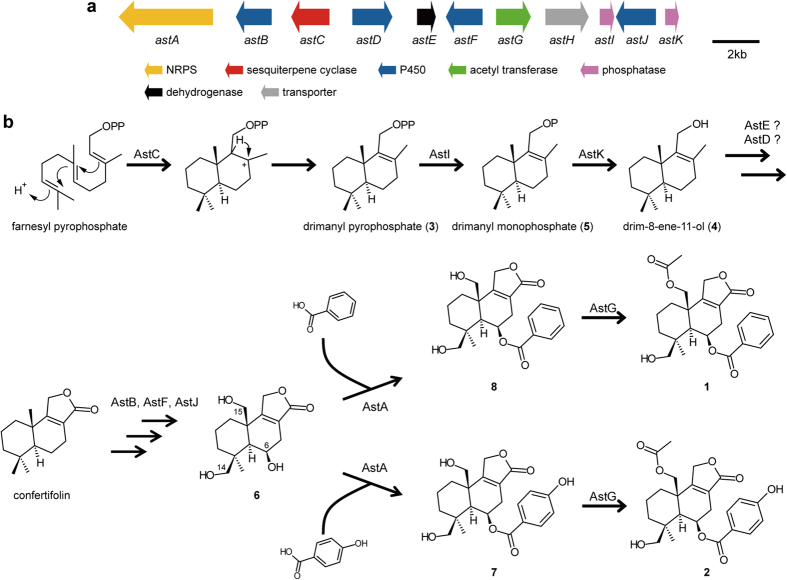
Summary of astellolide biosynthesis. (**a**) Schematic representation of the astellolide biosynthetic gene cluster in *A. oryzae*. (**b**) Proposed astellolide biosynthetic pathway.

Depyrophosphorylation of **3** is necessary for the biosynthesis of astellolides. A search in the Pfam database indicated that AstC belonged to a HAD-like hydrolase superfamily, some of whose members act also as phosphatases^19^. For example, HAD4 and HAD10 in *E. coli* are able to utilise isopentenyl pyrophosphate as a substrate for depyrophosphorylation^33^. Therefore, we speculated that AstC might have dephosphorylation activity. To test the effect of the His-tag on the phosphatase activity of AstC, we removed the tag and performed the enzymatic assay. We observed that AstC could not depyrophosphorylate **3** (Supplementary Fig. 6), hence concluding that AstC possessed only cyclisation activity (Fig. 6). Moreover, the experiment suggested the presence of unknown enzymes catalysing the depyrophosphorylation of **3**. Given that the astellolide biosynthetic gene cluster contained two other HAD-like hydrolase genes (*astI* and *astK*), we tested the pyrophosphate release activity of their products, AstI and AstK. We found that AstI catalysed the dephosphorylation of **3** into **5**, followed by dephosphorylation of **5** into **4** by AstK (Fig. 6).

Further investigation of the molecular basis of astellolide biosynthesis revealed that AstA was involved in catalysing ester bond formation (Fig. 6). In general, the NRPS A-domain activates the substrate by forming an acyl-adenylate intermediate, which it then transfers to the T -domain to yield a thioester-linked product^34^. In the case of multi-module NRPS, the C-domain catalyses the condensation (usually amide bond formation) between the two substrates tethered to upstream donor and downstream acceptor modules, and shows selectivity for the acceptor substrate^35^. In contrast, in single-module NRPS, the mechanism defining the acceptor substrate is not well understood. In this study, in which AstA catalyses ester bond formation between **3** and aryl acid, the C-domain of AstA may use **6** as a nucleophile to attack the thioester bond of an enzyme-tethered aryl acid. Although C-domains, such as Fum14p^36^, SgcC5^37^, and CrpD-M2^38^ have been reported to catalyse ester bond formation, AstA is the first example of NRPS employing this mechanism in terpenoids (Fig. 6).

Recently, we showed that **2** (but not **1**) had antiproliferative activity against several tumour cell lines^12^. This result indicates that modification of the benzoate moiety of astellolides affects this activity. The amino acid-activating A-domains generally have high substrate specificity. However, in some instances, they display broad substrate specificity, as with the A1-domain of AFUA_6g12080, which recognises and activates carboxylic acids^39^. To this end, the A-domain of AstA exhibits similar substrate preference for both BA and 4HBA. It may be possible to produce a variety of aryl-substituted astellolide derivatives by examining the substrate specificity of AstA; this possibility is currently under investigation.

In summary, we identified the astellolide biosynthetic gene cluster of *A. oryzae* using the *cclA* disruption strain. In light of the results obtained by gene disruption, metabolite analysis of gene disruptants, and biochemical analysis of purified enzymes, we proposed a new biosynthetic pathway (Fig. 6). Importantly, we characterised a novel drimane-type sesquiterpene biosynthetic machinery composed of AstC, AstI, and AstK, and containing a HAD-like hydrolase domain. Moreover, we identified AstA as a unique NRPS responsible for catalysing ester bond formation with terpenoids. Our findings provide new insight into the sesquiterpene biosynthetic machinery.

## Methods

### Strains and transformation of *A. oryzae*

All *A. oryzae* strains used in this study were derived from *A. oryzae* RIB40 and are listed in Supplementary Table 3. RkuptrP2-1ΔAF/P was used as a control for the *cclA* disruption strain (Δ*cclA*). To generate *ast* disruption strains in a Δ*cclA* background, *pyrG* was deleted from the Δ*cclA* strain by *pyrG*-marker recycling^40^. Gene disruption and *pyrG* marker recycling cassettes were constructed by fusion PCR (see Supplementary Methods). The primers used for PCR are listed in Supplementary Table 4.

*A. oryzae* transformation was performed as described previously^41^. Czapek-Dox (CD) minimal medium (0.2% NaNO_3_, 0.1% KH_2_PO_4_, 0.05% KCl, 0.05% MgSO_4_ · 7H_2_O, 0.001% FeSO_4_ · 7H_2_O, 3% glucose, 2% agar) containing 1.2 M sorbitol was used as selective medium for transformation. CD minimal medium containing 1.2 M sorbitol and 15 mM uridine was used to regenerate mycelia from the protoplast during *pyrG* marker recycling transformation. Thereafter, positive selection of *pyrG*-deficient strains was performed using CD minimal medium containing 15 mM uridine and 5-fluoroorotic acid (2 mg/mL; Sigma, St. Louis, MO, USA).

### Total RNA preparation and DNA microarray analysis

*A. oryzae* transformants were inoculated onto Czapek yeast (autolysate) extract agar (CYA) medium (3% sucrose, 0.5% yeast extract, 0.3% NaNO_3_, 0.1% K_2_HPO_4_, 0.05% KCl, 0.05% MgSO_4_·7H_2_O, 0.001% FeSO_4_·7H_2_O, and 2% agar, pH 6.0) at 30 °C. After five days of cultivation, the mycelia of each transformant were collected and total RNA was extracted using the ISOGEN RNA Extraction Reagent (Nippon Gene, Tokyo, Japan). To remove genomic DNA, RNA samples were treated with DNase I (Takara Bio, Otsu, Japan). Samples were further purified using the RNeasy Mini Kit (Qiagen, Tokyo, Japan) and RNA quality was evaluated by agarose gel electrophoresis and ultraviolet spectrophotometry. DNA microarray analysis was performed as described previously^42^.

### qRT-PCR

Total RNA was reverse-transcribed using random hexamers and the PrimeScript RT reagent kit (Takara Bio). Gene expression was quantitatively assessed by qRT-PCR using SYBR Premix Ex Taq II (Takara Bio) on a Mx3000p cycler (Stratagene, Cedar Creek, TX, USA). Primers used for PCR are listed in Supplementary Table 5. All reactions were performed in duplicate on at least three independent RNA preparations. Data were analysed using the relative standard curve method with histone 2B as the reference gene.

### Metabolite extraction and analysis

*A. oryzae* transformants were inoculated onto CYA plates and incubated for seven days at 30 °C. Metabolite extraction and LC/ESI-MS analysis were performed as described previously^12^. Briefly, 6-mm-diameter plugs were removed from plate cultures and 10 plugs from each transformant were used for extraction. The plugs were extracted with ethyl acetate (2 mL). The extracts (1.6 mL) were dried in a vacuum centrifuge, dissolved in acetonitrile (160 μL), and subjected to LC/ESI-MS analysis.

### Isolation and structure elucidation of 6, 7, and 8

The Δ*cclA* Δ*astA* and Δ*cclA* Δ*astG* double disruption strains were cultured at 30 °C for 7 days on CYA plates (1 L) and used for the isolation of **6**, and **7** and **8**, respectively. Isolation was performed as described previously^12^ except that UV detection was carried out at 220 nm. HRESI mass spectra were measured using a QSTAR Elite apparatus (Applied Biosystems/MDS SCIEX, Foster City, CA, USA), with a mixture of CsI (*m*/*z* 132.9054) and sex pheromone inhibitor iPD1 (*m*/*z* 829.5398) (Applied Biosystems/MDS SCIEX) as the calibration standard. ^1^H-NMR (500 MHz), ^13^C-NMR (125 MHz), correlation, heteronuclear single quantum coherence, and heteronuclear multiple bond correlation spectra were recorded in DMSO-*d*_6_ at room temperature using a Bruker AVANCE 500 spectrometer (Bruker, Billerica, MA, USA). The ^1^H, ^13^C, and 2D NMR spectral data are presented in Supplementary Table 2 and Supplementary Fig. 3.

### Cloning, expression, and purification of AstA, AstC, AstI, and AstK

Details are provided in Supplementary Methods.

### *In vitro* AstA enzyme assay

The reaction was performed as described previously^43^ with minor modifications. The reaction mixture (100 μL) consisted of 50 mM Tris-HCl (pH 8.0), 10 mM MgCl_2_, 5 mM ATP, 2 μM Sfp from *Bacillus subtilis*^44^ (New England Biolabs, Beverly, MA, USA), 200 μM CoA, 1 mM DTT, 200 μM **6**, 200 μM BA or 4HBA, and 1 μM purified AstA protein. Following incubation of the mixture at 30 °C for 1 h, the reaction was terminated by adding ethyl acetate (1 mL). The mixture was agitated vigorously and then centrifuged for 10 min at 16,000 × *g*. The supernatant ethyl acetate layer (900 μL) was dried in a vacuum centrifuge at 25 °C. The residue was dissolved in acetonitrile (90 μL) and subjected to LC/ESI-MS analysis. A control reaction mixture with boiled AstA was prepared and treated under the same conditions. To determine the kinetic parameters of the A-domain in AstA, inorganic pyrophosphate released by the enzymatic reaction was measured using the EnzChek Pyrophosphate Assay Kit (Thermo Fisher Scientific, Waltham, MA, USA). The reaction mixture (100 μL) consisted of 50 mM Tris-HCl (pH 7.4), 5 mM MgCl_2_, 2.5 mM ATP, 1 mM DTT, 0.2 mM 2-amino-6-mercapto-7-methylpurine ribonucleoside, 1 unit/mL purine nucleoside phosphorylase, 0.03 units/mL inorganic pyrophosphatase, the substrate (BA or 4HBA) at a concentration of 1.25 to 10 μM, and 2 μM purified AstA protein. Each reaction was initiated by the addition of substrate and monitored at 360 nm on an Infinite M200 microplate reader (Tecan, Grödig, Austria). Initial velocities were calculated using the standard curve created with the pyrophosphate standard from the kit.

### *In vitro* enzymatic assay for AstC, AstI, and AstK

For the AstC enzymatic assay, the reaction mixture (500 μL) contained 50 mM Tris-HCl (pH 8.0), 10 mM MgCl_2_, 5 mM DTT, 100 μM FPP, and 1 μM purified AstC protein. Following incubation at 30 °C for 1 h, alkaline phosphatase (10 μL, 5 units, Takara Bio) was added and the incubation was continued at 37 °C for 1 h. For AstI and AstK assays, alkaline phosphatise was replaced by 1 μM purified AstI, AstK, or both and the reaction was incubated at 30 °C for 1 h. The reaction was terminated by adding 0.5 M EDTA (100 μL, pH 8.0). The reaction mixture was extracted with ethyl acetate (250 μL) and subjected to GC-MS analyses or filtered through a 0.45-μm filter (Merck Millipore, Billerica, MA, USA) prior to HPLC analyses. This was performed on a Shimadzu Prominence LC solution system (Shimadzu, Kyoto, Japan) using a COSMOSIL 5C_18_-MS-II column (4.6 × 150 mm; Nacalai Tesque, Kyoto, Japan) with 25 mM NH_4_HCO_3_ in water/acetonitrile (75:25, v/v) as the mobile phase at a flow rate of 1 mL min^−1^. UV detection was performed at 210 nm. GC-MS analysis was carried out on an Agilent 5975 GC-MSD system equipped with a HP-5MS UI column (30 × 0.25 mm × 0.25 μm). GC conditions were as follows: oven temperature from 50 °C to 260 °C at 10 °C min^−1^; injector and detector temperature, 250 and 280 °C, respectively. The compound was identified by comparing its MS spectrum to that found in the Wiley 9th edition NIST11 (W9N11) mass spectral library. Pi released during the incubation was measured using a malachite green assay kit (Bioassay Systems, Hayward, CA, USA). Briefly, 1 μM purified AstI was incubated with the AstC reaction mixture, which was prepared as described above except for a different concentration of FPP (20 μM). After 30 min of incubation at 30 °C, the reaction was terminated by adding the malachite green reagent (4:1 v/v); 30 min later the concentration of Pi was measured at 620 nm on an Infinite M200 microplate reader (Tecan).

### Isolation and structure elucidation of the AstC reaction product

The reaction mixture (10 mL × 6 samples), containing 50 mM Tris-HCl buffer (pH 8.0), 0.1 mM MgCl_2_, 5 mM DTT, 260 μM FPP, and 1 μM purified AstC, was incubated at 30 °C for 1 h. Then, 0.5 M EDTA (pH 8.0, 2.5 mL) was added to terminate the reaction. The reaction mixture was applied to a Sep-Pak C_18_ cartridge (500 mg; Waters, Milford, MA, USA) pre-equilibrated with 25 mM NH_4_HCO_3_ in water. The cartridge was washed with 25 mM NH_4_HCO_3_ in water (5 mL) followed by 25 mM NH_4_HCO_3_ in water/acetonitrile (95:5, v/v; 5 mL). The product was eluted with 25 mM NH_4_HCO_3_ in water/acetonitrile (50:50, v/v; 3 mL). The eluent was then evaporated to remove acetonitrile and purified by reverse-phase HPLC. HPLC conditions were as follows: column, COSMOSIL 5C_18_-AR-II (20 × 250 mm) (Nacalai Tesque); flow rate, 4 mL min^−1^; solvent, 25 mM NH_4_HCO_3_ in water/acetonitrile (75:25, v/v). UV detection was performed at 210 nm. The UV-active fraction was evaporated under reduced pressure to precipitate the compound as a white powder.

## Additional Information

**How to cite this article**: Shinohara, Y. *et al*. Identification of a novel sesquiterpene biosynthetic machinery involved in astellolide biosynthesis. *Sci. Rep.* **6**, 32865; doi: 10.1038/srep32865 (2016).

## Supplementary Material

Supplementary Information

## Figures and Tables

**Table 1:** Organisation and putative functions of genes in the astellolide biosynthetic gene cluster.

Name	Original gene ID (revised gene ID)^a^	Size (aa)	Putative function	Protein homolog (species, NCBI accession No)	Identity/similarity (%)	Conserved domain (e-value)^b^
-	AO090026000587 (AORIB40_05904)	317	unknown	hypothetical protein (*A. flavus*, KJJ29440.1)	99/100	no conserved domain detected
-	AO090026000586 (AORIB40_05905)	730	ammonia lyase	aromatic amino acid lyase (*A. parasiticus*, KJK60308.1)	97/98	lyase_aromatic (3.6e-152)
AstA	AO090026000585 (AORIB40_05906)	1338	ester-bond forming NRPS	AMP-dependent synthetase/ligase (*P. expansum*, KGO43219)	40/55	AMP-binding (5.4e-62)
						PP-binding (6.2e-5)
						Condensation (1.3e-12)
AstB	AO090026000584 (AORIB40_05907)	513	cytochrome P450	cytochrome P450 (*A. niger*, XP_001391091.1)	50/68	cytochrome P450 (5.3e-63)
AstC	AO090026000582 (AORIB40_05908)	480	sesquiterpene cyclase	HAD-like hydrolase (*A. parasiticus*, KJK60343.1)	97/98	HAD-like hydrolase (2.8e-19)
AstD	AO090026000581 (AORIB40_05909)	512	cytochrome P450	hypothetical protein (*P. brasilianum*, CEJ62411.1)	62/75	cytochrome P450 (5.3e-73)
AstE	AO090026000580 (AORIB40_05910)	261	dehydrogenase	glucose dehydrogenase (*Citricoccus sp*., WP_010144002.1)	55/71	short-chain dehydrogenase (3.6e-50)
AstF	AO090026000579 (AORIB40_05911)	521	cytochrome P450	cytochrome P450 (*A. parasiticus*, KJK60355.1)	96/97	cytochrome P450 (7.2e-69)
AstG	AO090026000578 (AORIB40_05912)	471	acetyl transferase	hypothetical protein (*P. crustosum*, AGZ20197.1)	33/52	transferase (6.8e-24)
AstH	AO090026000587 (AORIB40_05913)	564	transporter	drug resistance transporter EmrB/QacA subfamily protein (*A. parasiticus*, KJK60349.1)	97/98	major facilitator superfamily transporter (7.9e-39)
AstI	AO090026000576 (AORIB40_05914)	201	phosphatase	HAD-like hydrolase (*A. parasiticus*, KJK60328.1)	96/98	HAD-like hydrolase (1.1e-28)
AstJ	AO090026000575 (AORIB40_05915)	507	cytochrome P450	cytochrome P450 (*A. parasiticus*, KJK60354.1)	57/72	cytochrome P450 (3.3e-67)
AstK	- (AORIB40_NS.05916)	196	phosphatase	phosphatase yihX, putative (*A. flavus*, XP_002379379.1)	99/99	HAD-like hydrolase (1.8e-29)
-	- (AORIB40_NS.05917)	187	oxidoreductase	oxidoreductase, putative (*A. flavus*, XP_002379380.1)	100/100	GFO_IDH_MocA (4.4e-15)
-	AO090026000574 (AORIB40_05918)	633	unknown	hypothetical protein (*A. flavus*, KOC17980.1)	97/97	no conserved domain detected

^a^Revised annotation information is available from NITE.

^b^Protein domain analysis was performed using the Pfam database (http://pfam.xfam.org/).
