# Supplementary material for: Identification of a novel sesquiterpene biosynthetic machinery involved in astellolide biosynthesis
Source: Sci Rep. 2016 Sep 15;6:32865. doi: 10.1038/srep32865 (PMC5024094; doi:10.1038/srep32865)
Supplement: Supplementary Information [file srep32865-s1.pdf]

Supplementary Information

Identification of a novel sesquiterpene biosynthetic machinery involved in astellolides biosynthesis

Yasutomo Shinohara[1], Shunji Takahashi[2], Hiroyuki Osada[2] and Yasuji Koyama[1]

[1]Noda Institute for Scientific Research, 399 Noda, Noda, Chiba 278-0037, Japan.

[2]Chemical Biology Research Group, RIKEN CSRS, 2-1 Hirosawa, Wako, Saitama 351-0198, Japan.

Supplementary Methods

Construction of the transformation cassette for gene disruption.

Gene disruption and *pyrG* marker recycling cassettes were constructed by fusion PCR. Briefly, the following three PCR fragments were amplified for gene disruption: 5'-region of the target gene (primers X-LU and X-LL), 3'-region (primers X-RU and X-RL), and auxotrophic marker *pyrG* (primers pyrG-U and pyrG-L). The *pyrG* marker recycling cassette was constructed following amplification of the following two fragments: 5'-upstream region of the *cclA* gene (primers cclA-LU and cclA-LL) and 3'-downstream region of the *cclA* gene (primers cclA-RU and cclA-RL). Each fragment was amplified from the genomic DNA of *A. oryzae* RIB40. Two or three amplified fragments were mixed and subjected to fusion PCR with nested primers X-LU-2 and X-RL-2, where "X" represents the name of each target gene. The resulting PCR products were purified and used as a transformation vector. The primers used for PCR are listed in Supplementary Table 4.

Cloning, expression, and purification of AstA, AstC, AstI, and AstK.

Escherichia coli BL21, expression vectors pColdI and pColdIV, and restriction enzymes were purchased from Takara Bio (Otsu, Japan). The *astA* gene fused with the C-terminal His tag, and the *astC*, *astI*, and *astK* genes were amplified using primers astA-L [5'-TCGCCATATGGACCTGGAGCCGTGGGGA-3' (*Nde*I site underlined)] and astA-His-R [5'-ACTGGTCGACTTA**GTGATGGTGGTGATGATG**TAAAACACTATCAGGAGAATCAAGA-3' (*Sal*I site underlined and His tag in bold)], astC-L [5'-TCGCCATATGACCAAGATCAACCCCTACA-3' (*Nde*I site underlined)] and astC-R [5'-ACTGGTCGACAATTCCAGCGCACTGGTTGGCT-3' (*Sal*I site underlined)], astI-L [5'-TCGCCATATGACACGTCAGAGTCACTATCAAGC-3' (*Nde*I site underlined)] and astI-R [5'-ACTGGTCGACTCAAATCCCAAGAAGATGTCCCCGCA-3' (*Sal*I site underlined)] and astK-L [5'-TCGCCATATGTGCACTACCTTCAAGGCTGCCA-3' (*Nde*I site underlined)] and astK-R [5'-ACTGCTGCAGCTAGCAGCCAACCAGCTGTCGT-3' (*Pst*I site underlined)], respectively. *A. oryzae* RIB40 cDNA was prepared as described above. The PCR products were purified using a gel extraction kit (Qiagen, Tokyo, Japan) and cloned into the pTA2 vector using TArget Clone Plus (Toyobo, Osaka, Japan) according to the manufacturer's instructions, to generate pTA2-*astA*His, pTA2-*astC*, pTA2-*astI*, and pTA2-*astK*. The fragments excised by *Nde*I and *Sal*I from pTA2-*astA*His, pTA2-*astC*, and pTA2-*astI* or by *Nde*I and *Pst*I from pTA2-*astK* were ligated into the *Nde*I/*Sal*I sites of pColdIV (*astA*) and pColdI (*astC* and *astI*) or into the *Nde*I/*Pst*I sites of pColdI (*astK*) to obtain

pColdIV-*astA*His, pColdI-*astC*, pColdI-*astI*, and pColdI-*astK*, respectively. The expression vectors were confirmed by DNA sequencing. The *E. coli* BL21 strain harbouring pColdIV-*astA*His, pColdI-*astI*, or pColdI-*astK* was grown at 37 °C in Luria-Bertani medium containing ampicillin (100 μg mL^{-1}). The *E. coli* BL21 strain harbouring pColdI-*astC* was grown at 37 °C in terrific broth containing ampicillin (100 μg mL^{-1}). When culture density reached OD_{600} 0.6–0.7, cells were kept at 15 °C for 30 min and 0.1 mM isopropyl β-D-thiogalactoside was added; the culture was then incubated for 20 h at 15 °C. Cells were harvested by centrifugation (10 min at 3,000 × *g*) and resuspended in 50 mM Tris-HCl (pH 8.0) containing 150 mM NaCl, 1 mM dithiothreitol (DTT), 10% glycerol, and 10 mM imidazole. Resuspended cells were disrupted by 10 × 30 s sonication cycles on ice. After centrifugation for 20 min at 16,000 × *g*, the supernatants were loaded onto HisTrap FF Columns (GE Healthcare, Buckinghamshire, UK) and washed with 50 mM Tris-HCl (pH 8.0) containing 0.5 M NaCl, 10% glycerol, and 20 (AstA) or 60 mM imidazole (AstC, AstI and AstK). The recombinant enzymes were finally eluted with 50 mM Tris-HCl (pH 8.0) containing 0.5 M NaCl, 10% glycerol, and 300 mM imidazole. The purified proteins were desalted and concentrated with Amicon 100-kDa (AstA), 10-kDa (AstC) and 3-kDa (AstI and AstK) filters, respectively (Merck Millipore, Billerica, MA, USA). To examine whether the His-tag affected AstC function, the tag was removed by treatment with Factor Xa (Merck Millipore) at 20 °C for 18 h, and then re-purified with HisTrap FF columns. Protein concentrations were determined using the Bradford method with bovine serum albumin as a standard.

```
AstC                                1 --MTKIN---PYKGILVELKDIVFTS-SSD-QIKLPINTFKSILCCGATAQYQCGKIN-RAQYYSRLARD 62
XP_006461126_Agaricus bisporus      1 ----MAPPQRPFTAIVFDIGDVLFQW-SATTKTSISPKTLRSILNCPTWFDYERGRLA-ENACYAAISQE 64
XP_001877382_Laccaria bicolor       1 --------------IIFDLGDVVFKW-SPETKTSISSRTLRDILSSPTWFNYERGQLA-EEECYQQIGEE 54
XP_007864346_Gloeophyllum trabeum   1 ----MVAAYRRFSTLICDIGDVLFTW-SSTTRTTISARTLKAMMTSPTWRLYERGHYS-QGQCYDQLSKD 64
XP_001391086_Aspergillus niger      1 MVVAVPVTSSPDRAILVELNHVIFKS-SFV-DVGIPENTYKSILCCGASTEYQCGKAG-LDQYYSRLSKD 67
XP_001217376_Aspergillus terreus    1 ----MAITKGPVKALILDFSNVLCSW-KPPSNVAVPPQILKMIMSSDIWHDYECGRYS-REDCYARVADR 64
XP_013943363_Trichoderma atroviride 1 MPHSTLTRPKSFHAIVLDLNGVLLSYGGSFPSSVLKPSQIKNVLDSPTWYDYECGKISSRQECYQRVSSE 70
CDM29845_Penicillium roqueforti     1 --MGKIIKRIPYKGVLIELKNIVLHS-SMQ-NIELPTNTLKSILYCGATVEYQCGRIT-EEQYFARLASD 65

AstC                               63 FA-LSLADVTALFDTVQATIRPEESFLAFLAELKSRFGEQLKLYAVANMSREDYAMLKSLPIDWSLFDGV 131
XP_006461126_Agaricus bisporus     65 FN-VNPDEVRDAFSQARDSLQANHDFISLIRELKAQANGRLRVYAMSNISLPDWEVLRMKPADWDIFDHV 133
XP_001877382_Laccaria bicolor      55 FN-LLSGEVRRAFDQARESLVADEALIDLIRDLKTQSDGRLRIFAMSNISPPDWAVLRTKPADWSIFDQV 123
XP_007864346_Gloeophyllum trabeum  65 FPPYEPEEIGRAMDEARDSLTSNDAMIALLRQLKSESNGQLRILAMSNISKPDYMFLRTKPADWDVFDTV 134
XP_001391086_Aspergillus niger     68 FD-TPKSQIHDMFSAINKTVQVDHGILACLARLKAHCRGTLAIYGACNMSCKDFEKVQGYSIDWNIFDGI 136
XP_001217376_Aspergillus terreus   65 FH-ISAADMEDTLKQARKSLQVHHETLLFIQQVKKDAGGELMVCGMTNTPRPEQDVMHSINAEYPVFDRI 133
XP_013943363_Trichoderma atroviride 71 FE-MDVDVFSDTLEQLTKTVKPHSEFIAAIKNIKAAFP-EIKVYGMSNISQPDYEFLKPMISSWGILDGF 138
CDM29845_Penicillium roqueforti    66 FR-HPQEEIKKAILAVRKSLCVNPKVVEALASMKAKSKGLFELYAVTNFSKEDYALVKFLGFDWSLFERV 134

AstC                              132 FLSADLGMRKPELRFFRHVLESISMKPEDTILVDNDTDNILCALSMGLKGILFGSTS-VPQALTNLLEYD 200
XP_006461126_Agaricus bisporus    134 FTSGAVGERKPNLAFYRHVIAATDLQPHQTIFVDDKLENVLSARSLGFTGIVFDEPSEVKRALRNLIG-D 202
XP_001877382_Laccaria bicolor     124 FTSGSAGERKPNLGFYEHVLAGTGVGPRQTIFVDDKLENVISARSLGFHGIVFDSPEPVKRALRNLTG-D 192
XP_007864346_Gloeophyllum trabeum 135 FTSSDAGERKPNLGYYRYVVEKGAIDPHSTIFIDDKLENVLVARSLGMYGIVFDDQDKVMRALRNLLG-D 203
XP_001391086_Aspergillus niger    137 FISGDMGISKPELRFFSHILDRLQLAPSEVIVVDHNTDNVLTAISMGMSAVLANSPDDVQRSLVNYIERN 206
XP_001217376_Aspergillus terreus  134 YISGLMGMRKPSICFYQRVMEEIGLSGDAIMFIDDKLENVIAAQSVGIRGVLFQSQQDLRRVVLNFLG-D 202
XP_013943363_Trichoderma atroviride 139 QASGQAGVRKPDNASYITFLQKFELDSARCVFIDDSVENTVAASALGFKGVKYSNPIEVERTLWNLLG-S 207
CDM29845_Penicillium roqueforti   135 FVSSDIGMQKPELRFYQHVLNQIGLSSEQVILVDDDTSNLLAAMSMGMQGVMPSDYS-LYRSILNFVDID 203

AstC                              201 HISRAEQFLRSHAKSLHSVTHTG-----VTIRENFAQLLILEATGDIDLVEL-EYHPTTWNYFIGTPVLT 264
XP_006461126_Agaricus bisporus    203 PVQRGGEFLVRNAGKLGSITRTTAKHESIPLDENFAQLLILEITGNRALVNL-VEHPQTWNFFQGKGQLT 271
XP_001877382_Laccaria bicolor     193 PLARGQAFLHENAGNLVSVTENSDNHEAVLLQENFAQLLILEVIGDANLVNL-VEHPRTWSFFQGKGQLT 261
XP_007864346_Gloeophyllum trabeum 204 PIQRGWSFMRSHAGHLESVTNTG-----VLVPETFAQLLILDVTQDRNLVALPKDCPRRWNFFSGKPILT 268
XP_001391086_Aspergillus niger    207 PTERGRKFLERNAKNMHSVTHTG-----VLIRENFAQLMILEASGDSTLVDI-KPHATTWNYFIDKPVLT 270
XP_001217376_Aspergillus terreus  203 PVHRGLQFLAANAKKMDSVTNTG-----DTIQDNFAQLLILELAQDRELVKL-QAGKRTWNYFIGPPKLT 266
XP_013943363_Trichoderma atroviride 208 PVDRGMEYMERNAKKMMLELSTG-----GEQPDNFSQFIILELTQDERLIKLERKRGPTWNYFHHSNTFN 272
CDM29845_Penicillium roqueforti   204 PIGRGTRYLHENAQKHHSFTHTG-----VPVKENFTQLLILELTGDRSLIDI-GSHRTTWNLFIVTPVLT 267

AstC                              265 QTEFPHDLDTTSLATTVLD-RPKDIANEIMDEMLKYRSDDDLMLTFFTDFKNRVDPVVCCNVLSLFYKYG 333
XP_006461126_Agaricus bisporus    272 TEEFPFDLDTTSLGLTILK-RSREIADSVMDEMLEYVDPDGIIQTYFDHRRPRFDPVVCVNALSLFYAYG 340
XP_001877382_Laccaria bicolor     262 TEEFPFDLDTTSLGLTVMK-RDKAVANSVMNEMLEYVDHDGIIQTYFDHRRPRFDPVVCVNALTLFYTHG 330
XP_007864346_Gloeophyllum trabeum 269 TEVFPDDLDTTSLGLTIFE-TDEAVAQSVIDEMLEYVTPDGIVQTYFDHTRPRFDPVVCVNVLSLFYSHN 337
XP_001391086_Aspergillus niger    271 QKNFPDDLDTTSLGLTITN-ATPEVANQVLNKILHYRTYDGLIMTFFTDFKNRVDPVVCCNVLSLFYQYG 339
XP_001217376_Aspergillus terreus  267 TATFPDDMDTTSMALSVLP-VAEDVVSSVLDEMLKFVTDDGIFMTYFDSSRPRVDPVVCINVLGVFCRHN 335
XP_013943363_Trichoderma atroviride 273 GTTYSDDCDTTSYAMCTLDDIPAHEKEAAMDIILNNLSPDNLPLCWFNKNRPRLCHGIIANAFRFFALSG 342
CDM29845_Penicillium roqueforti   268 QADFPDDMDTTSLGITILN-RPTHVANLVMDKMLQYRTSDGLMQTFFTDFKKRVDPIVCCNILNIFYQYG 336

AstC                              334 RGHELHHTLAWVRQVLIRRAYINGTAFYPMPEAFLYFFFRFLQHIT-HLPQLYDGLKVLLKERLQERVGV 402
XP_006461126_Agaricus bisporus    341 RGEQLRSTLTWVHEVLLNRAYLDGTRYYETAECFLYFMSRLLATSG--DPDLHSLLKPLLKERVQERIGA 408
XP_001877382_Laccaria bicolor     331 RGSELSRTLQWIHKVLLNRAYLDGTRYYQTAECFLFFLSRLLASSE--DRELHALLKPLLRERIQERIGV 398
XP_007864346_Gloeophyllum trabeum 338 RKQELRGTLIWVREVLLNRAYLDGTRYYASAECFLYFLSRLLRKAE--DHELNDLLYNLLKERVQERIGV 405
XP_001391086_Aspergillus niger    340 RGEEVSDTFDWVQLVLRRRAYIHGTAFYPSPEAFLFFFSRLLRRLESPPTPTYNELEQLLRERVAERIGV 409
XP_001217376_Aspergillus terreus  336 RERDVLPTFHWIRDILINRAYLSGTRYYPSPDLFLFFLARLCLAVR--NQSLREQLVLPLVDRLRERVGA 403
XP_013943363_Trichoderma atroviride 343 QGHKLAHTYLFLCRLLRTKAYELGSRYYENIDYMPYILSNLCSRRP--TDPSLAEMRELLKKEIQDRSGC 410
CDM29845_Penicillium roqueforti   337 WGNELSETFDWVYQVLQTRTYIHGSAFYPLPEAFFFFLSRMMLRLKNHRPCVYIRMRGLLIKRLEERLSV 406

AstC                              403 PVDPISLSMRLIACNGVGIH-DRMGLNALLSMQNPDGSWDLGTMYHYASKRLPIGNQGVSTAMAIKAIKQ 471
XP_006461126_Agaricus bisporus    409 DGDSLALAMRILACDFVGIR-DEVDLRTLLTLQCEDGGWEVGWMYKYGSSGISIGNRGLATALAIKAVDT 477
XP_001877382_Laccaria bicolor     399 EGDSIALAMRILVCDFVGLR-DEIDLRSLLPLQCEDGGWEIGWIYKYGSSGLRIGNRGLTTALALNAL-- 465
XP_007864346_Gloeophyllum trabeum 406 EGDALALAMRILTCAAVGIR-DDVDMRTLLPLQCEDGGWELGWIYKYGSSGVNIGNRGLATAFAIKAIEA 474
XP_001391086_Aspergillus niger    410 PVDAISLAMRLLVCHQVGMR-DTLGLEMLLSMQQPDGGWPLGTIYHYASKKQAIGNRGVSTALAVQAIDV 478
XP_001217376_Aspergillus terreus  404 PGEAVSLAARILACRSFGID-SARDMDSLRGKQCEDGGWPVEWVYRFASFGLNVGNRGLATAFAVRALES 472
XP_013943363_Trichoderma atroviride 411 DSDVLGAALRTLSAQAMGVPYAKRDVQVLLESQQLDGGWNRVWLFKYGKEDIKVGSRGVITAMAVKALRQ 480
CDM29845_Penicillium roqueforti   407 PVDAASLAMRLIVCHQVGVR-HVSGLKFLLSMQEPDGGWEIGTLYQYYSKRLWLGNRGTSTALALDAIRR 475

AstC                              472 CQANQCAGI------------------------------------------------------------- 480
XP_006461126_Agaricus bisporus    478 MFQPQIRFSESPTDTLVENAIHKRRPSFSEKFLGKRPRSGSFRKPLQWILQGSKLRKSVEIGS 540
XP_001877382_Laccaria bicolor     465 --------------------------------------------------------------- 465
XP_007864346_Gloeophyllum trabeum 475 LEDMPASPPTSGTTTPVK---HEEEAPRRVKRSSSRIRNHAVQAHF----------------- 517
XP_001391086_Aspergillus niger    479 CSQWKKSPNGHPKATVYTRTERHYGSP------------------------------------ 505
XP_001217376_Aspergillus terreus  473 PYGESAVKVMRRIV------------------------------------------------- 486
XP_013943363_Trichoderma atroviride 481 YYADEE--------------------------------------------------------- 486
CDM29845_Penicillium roqueforti   476 CQPWLAHRF------------------------------------------------------ 484
```

Supplementary Figure 1. Multiple sequence alignment of AstC and its orthologous proteins. Sequences were aligned using ClustalW. The conserved DxDTT motif in Class II terpene cyclase and QW motif are highlighted in red characters. The conserved DDxxD/E motif in Class I terpene cyclase and its region are highlighted in bold and underlined, respectively.

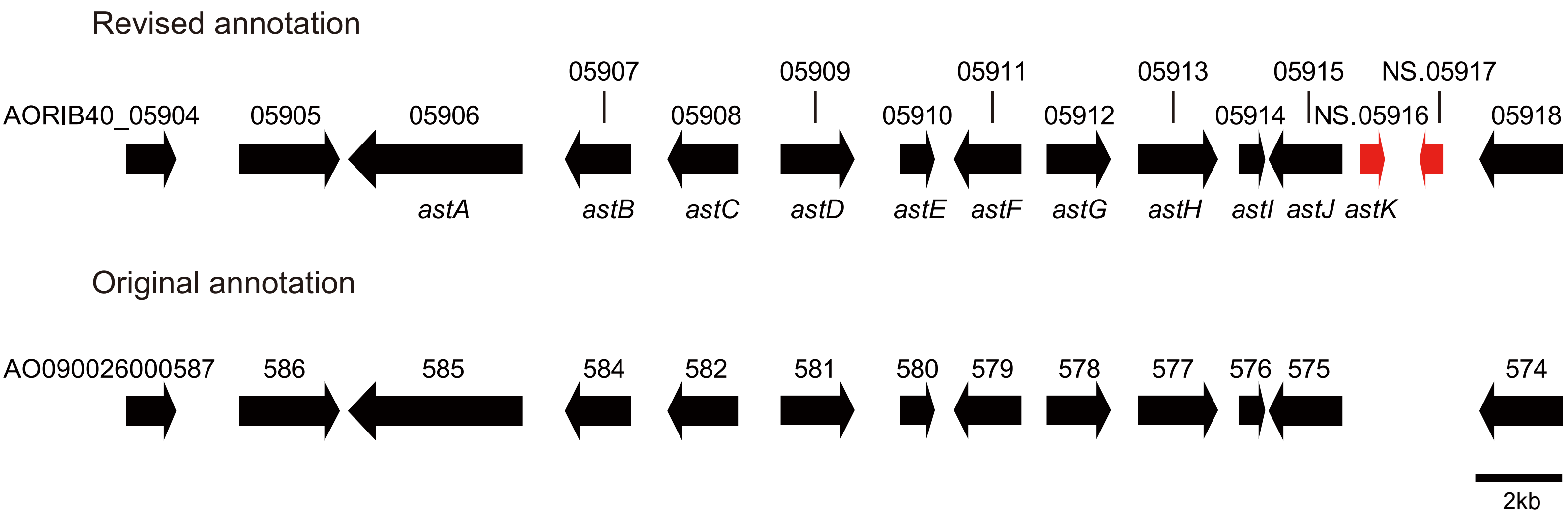

Gene name	revised annotation Locus Gene ID	Position Start	End	Dir	Gene bp	Protein aa	original annotation Locus Gene ID	Position Start	End	Dir	Gene bp	Protein aa
	AORIB40_05904	1638037	1639172	R	1136	317	AO090026000587	1638037	1639172	R	1136	317
	AORIB40_05905	1634259	1636578	R	2320	730	AO090026000586	1634259	1636578	R	2320	730
astA	AORIB40_05906	1630069	1634085	F	4017	1338	AO090026000585	1630069	1634085	F	4017	1338
astB	AORIB40_05907	1627556	1629097	F	1542	513	AO090026000584	1627556	1629097	F	1542	513
astC	AORIB40_05908	1625110	1626740	F	1631	480	AO090026000582	1625110	1626740	F	1631	480
astD	AORIB40_05909	1622434	1624148	R	1715	512	AO090026000581	1622434	1624148	R	1715	512
astE	AORIB40_05910	1620585	1621370	R	786	261	AO090026000580	1620585	1621370	R	786	261
astF	AORIB40_05911	1618599	1620164	F	1566	521	AO090026000579	1618599	1620164	F	1566	521
astG	AORIB40_05912	1616505	1618010	R	1506	471	AO090026000578	1616505	1618010	R	1506	471
astH	AORIB40_05913	1614075	1615892	R	1818	564	AO090026000577	1614075	1615892	R	1818	564
astI	AORIB40_05914	1612969	1613574	R	606	201	AO090026000576	1612969	1613574	R	606	201
astJ	AORIB40_05915	1611203	1612908	F	1706	507	AO090026000575	1611203	1612908	F	1706	507
astK	**AORIB40_NS.05916**	**1610213**	**1610803**	**R**	**591**	**196**	-					
	**AORIB40_NS.05917**	**1608890**	**1609453**	**F**	**564**	**187**	-					
	AORIB40_05918	1606152	1608053	F	1902	633	AO090026000574	1606152	1608053	F	1902	633

Supplementary Figure 2. Comparison of gene organisation between revised and original annotation in the astellolide biosynthetic gene cluster. Revised and original annotation information were obtained from NITE and *Aspergillus* genome database (AspGD; http://www.aspgd.org/), respectively. Genes that were added in the revised annotation are indicated in red.

a

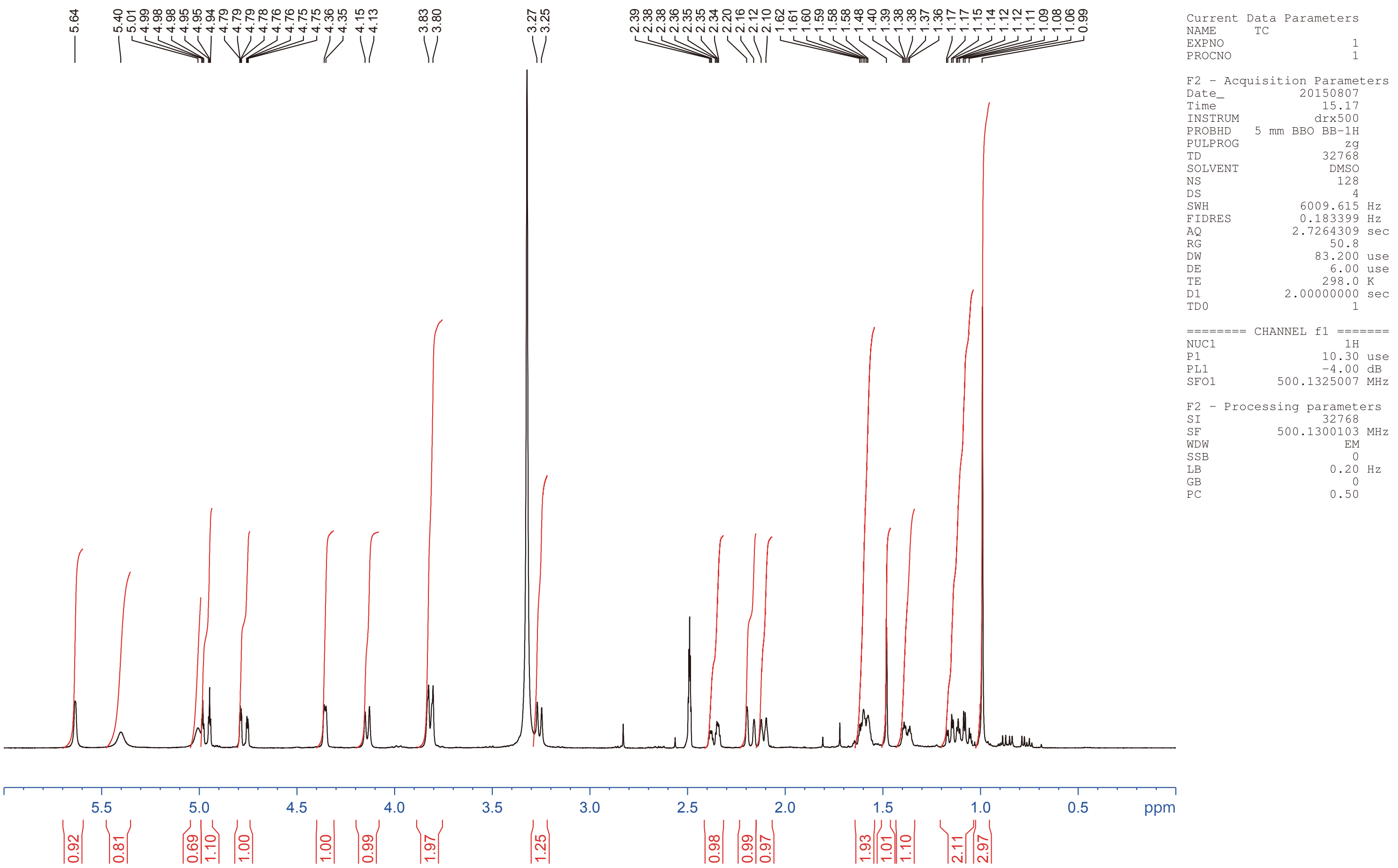
5.64
5.40 5.01 4.99 4.98 4.98 4.95 4.95 4.94 4.79 4.79 4.78 4.76 4.76 4.75 4.75 4.36 4.35 4.15 4.13
3.83 3.80
3.27 3.25
2.39 2.38 2.38 2.36 2.35 2.35 2.34 2.20 2.16 2.12 2.10 1.62 1.61 1.60 1.59 1.58 1.58 1.48 1.40 1.39 1.38 1.38 1.37 1.36 1.17 1.17 1.15 1.14 1.12 1.12 1.11 1.09 1.08 1.06 0.99
0.92 0.81 0.69 1.10 1.00 1.00 0.99 1.97 1.25 0.98 0.99 0.97 1.93 1.01 1.10 2.11 2.97
5.5 5.0 4.5 4.0 3.5 3.0 2.5 2.0 1.5 1.0 0.5 ppm
Current Data Parameters
NAME TC
EXPNO 1
PROCNO 1
F2 - Acquisition Parameters
Date_ 20150807
Time 15.17
INSTRUM drx500
PROBHD 5 mm BBO BB-1H
PULPROG zg
TD 32768
SOLVENT DMSO
NS 128
DS 4
SWH 6009.615 Hz
FIDRES 0.183399 Hz
AQ 2.7264309 sec
RG 50.8
DW 83.200 use
DE 6.00 use
TE 298.0 K
D1 2.00000000 sec
TD0 1
======== CHANNEL f1 =======
NUC1 1H
P1 10.30 use
PL1 -4.00 dB
SFO1 500.1325007 MHz
F2 - Processing parameters
SI 32768
SF 500.1300103 MHz
WDW EM
SSB 0
LB 0.20 Hz
GB 0
PC 0.50

b

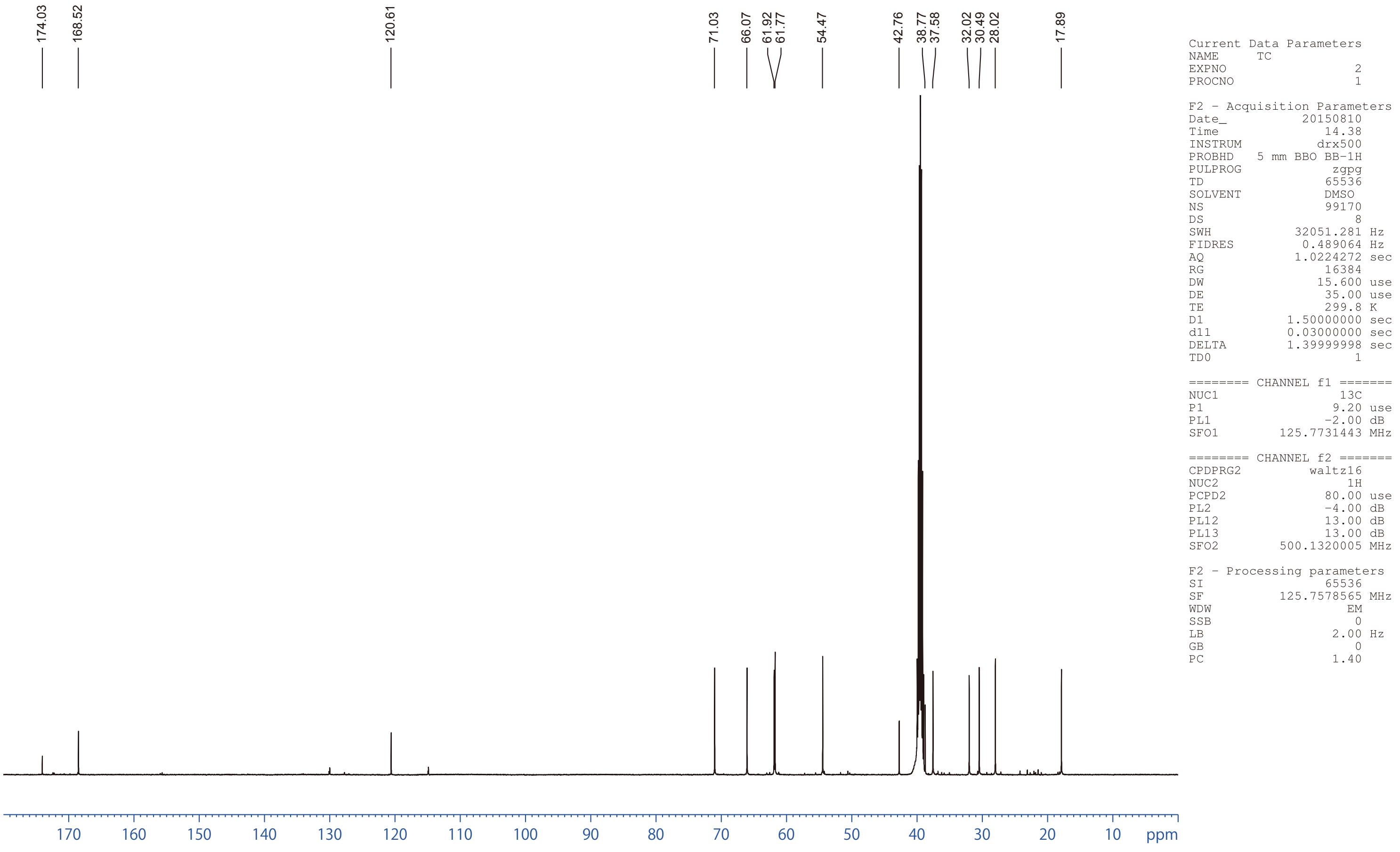
174.03 168.52 120.61 71.03 66.07 61.92 61.77 54.47 42.76 38.77 37.58 32.02 30.49 28.02 17.89
170 160 150 140 130 120 110 100 90 80 70 60 50 40 30 20 10 ppm
Current Data Parameters
NAME TC
EXPNO 2
PROCNO 1
F2 - Acquisition Parameters
Date_ 20150810
Time 14.38
INSTRUM drx500
PROBHD 5 mm BBO BB-1H
PULPROG zgpg
TD 65536
SOLVENT DMSO
NS 99170
DS 8
SWH 32051.281 Hz
FIDRES 0.489064 Hz
AQ 1.0224272 sec
RG 16384
DW 15.600 use
DE 35.00 use
TE 299.8 K
D1 1.50000000 sec
d11 0.03000000 sec
DELTA 1.39999998 sec
TD0 1
======== CHANNEL f1 =======
NUC1 13C
P1 9.20 use
PL1 -2.00 dB
SFO1 125.7731443 MHz
======== CHANNEL f2 =======
CPDPRG2 waltz16
NUC2 1H
PCPD2 80.00 use
PL2 -4.00 dB
PL12 13.00 dB
PL13 13.00 dB
SFO2 500.1320005 MHz
F2 - Processing parameters
SI 65536
SF 125.7578565 MHz
WDW EM
SSB 0
LB 2.00 Hz
GB 0
PC 1.40

c

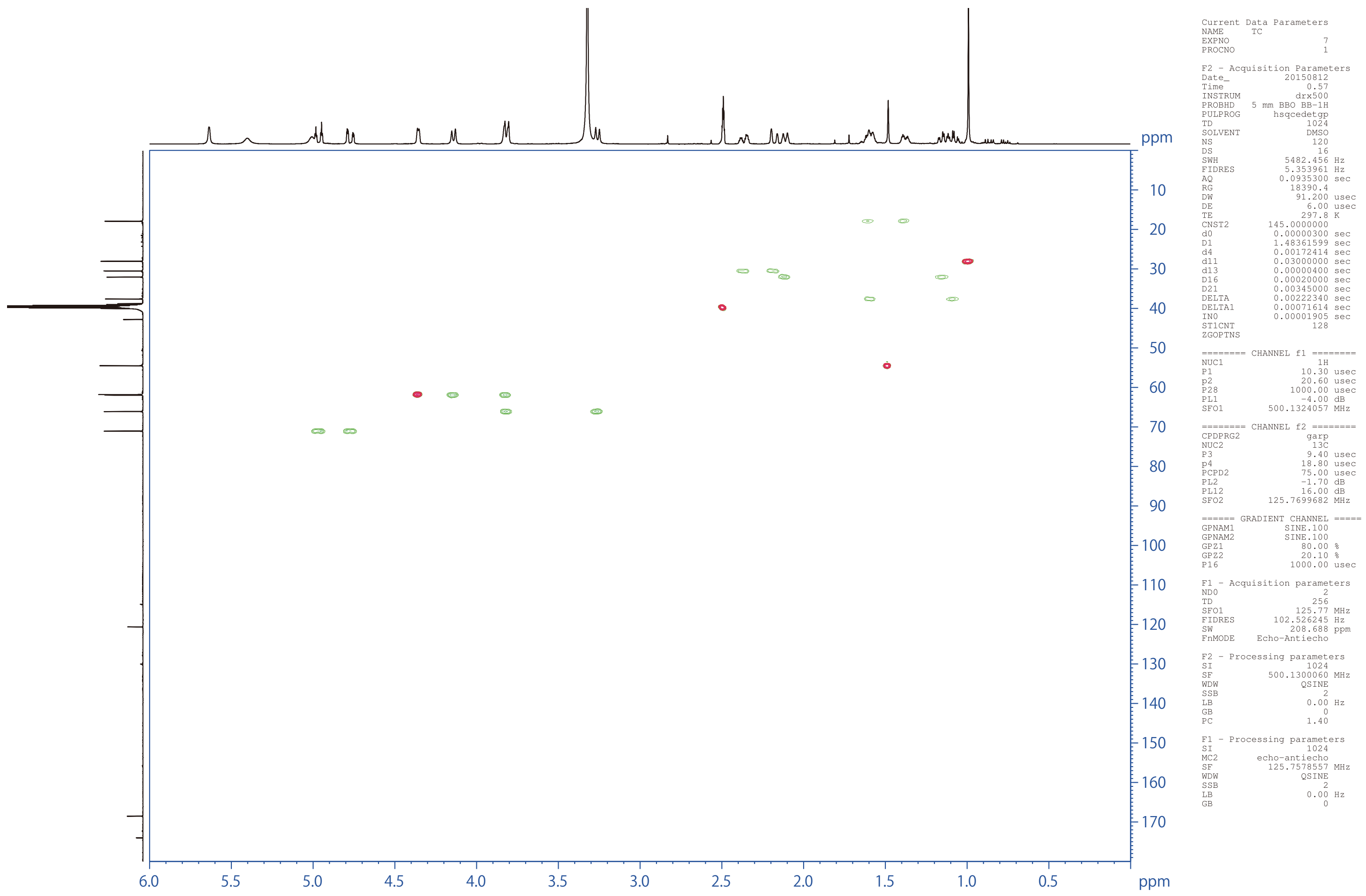

Current Data Parameters
NAME TC
EXPNO 7
PROCNO 1
F2 - Acquisition Parameters
Date_ 20150812
Time 0.57
INSTRUM drx500
PROBHD 5 mm BBO BB-1H
PULPROG hsqcedetgp
TD 1024
SOLVENT DMSO
NS 120
DS 16
SWH 5482.456 Hz
FIDRES 5.353961 Hz
AQ 0.0935300 sec
RG 18390.4
DW 91.200 usec
DE 6.00 usec
TE 297.8 K
CNST2 145.0000000
d0 0.00000300 sec
D1 1.48361599 sec
d4 0.00172414 sec
d11 0.03000000 sec
d13 0.00000400 sec
D16 0.00020000 sec
D21 0.00345000 sec
DELTA 0.00222340 sec
DELTA1 0.00071614 sec
IN0 0.00001905 sec
ST1CNT 128
ZGOPTNS
======== CHANNEL f1 ========
NUC1 1H
P1 10.30 usec
p2 20.60 usec
P28 1000.00 usec
PL1 -4.00 dB
SFO1 500.1324057 MHz
======== CHANNEL f2 ========
CPDPRG2 garp
NUC2 13C
P3 9.40 usec
p4 18.80 usec
PCPD2 75.00 usec
PL2 -1.70 dB
PL12 16.00 dB
SFO2 125.7699682 MHz
====== GRADIENT CHANNEL =====
GPNAM1 SINE.100
GPNAM2 SINE.100
GPZ1 80.00 %
GPZ2 20.10 %
P16 1000.00 usec
F1 - Acquisition parameters
ND0 2
TD 256
SFO1 125.77 MHz
FIDRES 102.526245 Hz
SW 208.688 ppm
FnMODE Echo-Antiecho
F2 - Processing parameters
SI 1024
SF 500.1300060 MHz
WDW QSINE
SSB 2
LB 0.00 Hz
GB 0
PC 1.40
F1 - Processing parameters
SI 1024
MC2 echo-antiecho
SF 125.7578557 MHz
WDW QSINE
SSB 2
LB 0.00 Hz
GB 0
ppm
ppm

d

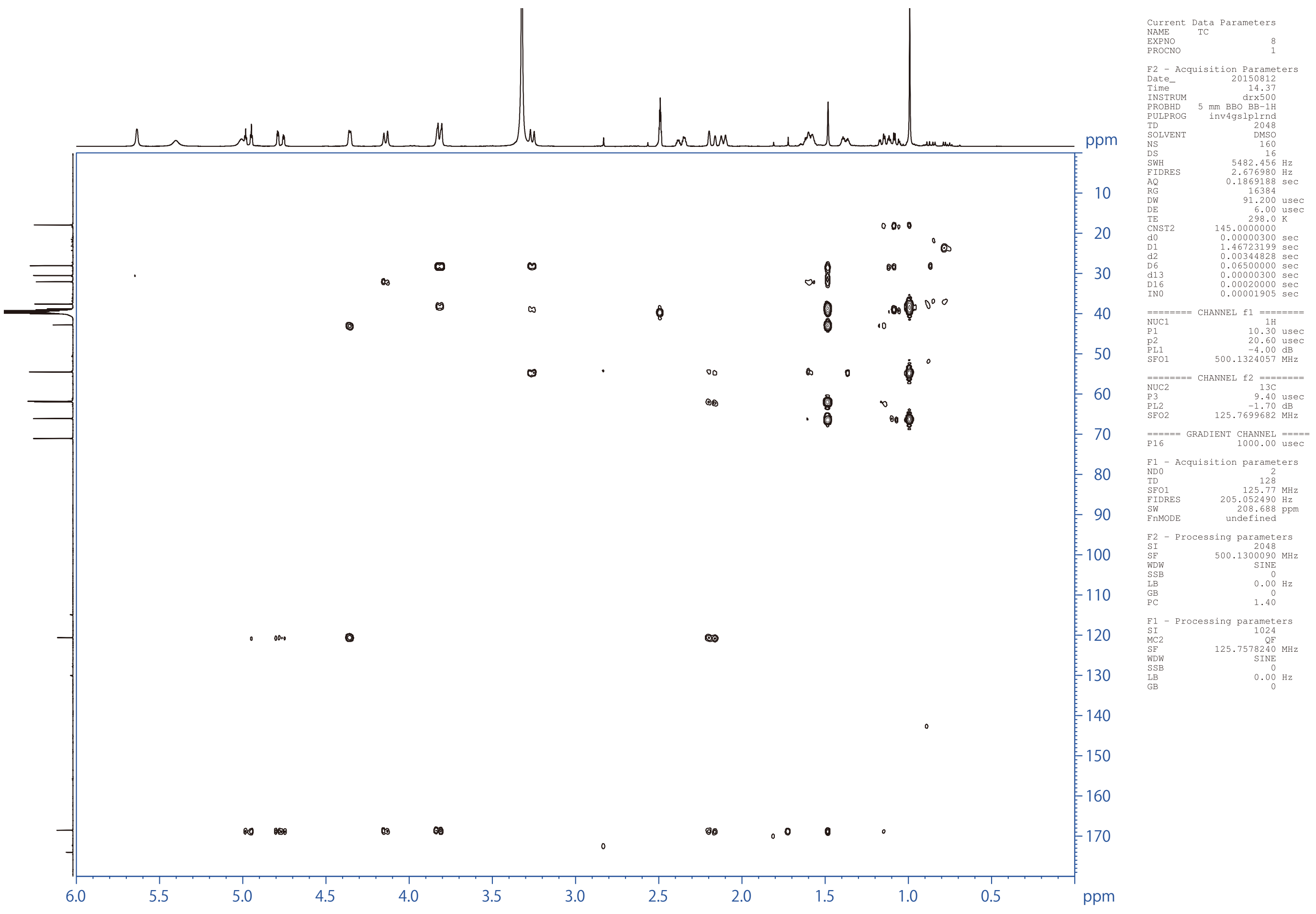

Current Data Parameters
NAME TC
EXPNO 8
PROCNO 1
F2 - Acquisition Parameters
Date_ 20150812
Time 14.37
INSTRUM drx500
PROBHD 5 mm BBO BB-1H
PULPROG inv4gslplrnd
TD 2048
SOLVENT DMSO
NS 160
DS 16
SWH 5482.456 Hz
FIDRES 2.676980 Hz
AQ 0.1869188 sec
RG 16384
DW 91.200 usec
DE 6.00 usec
TE 298.0 K
CNST2 145.0000000
d0 0.00000300 sec
D1 1.46723199 sec
d2 0.00344828 sec
D6 0.06500000 sec
d13 0.00000300 sec
D16 0.00020000 sec
IN0 0.00001905 sec
======== CHANNEL f1 ========
NUC1 1H
P1 10.30 usec
p2 20.60 usec
PL1 -4.00 dB
SFO1 500.1324057 MHz
======== CHANNEL f2 ========
NUC2 13C
P3 9.40 usec
PL2 -1.70 dB
SFO2 125.7699682 MHz
====== GRADIENT CHANNEL =====
P16 1000.00 usec
F1 - Acquisition parameters
ND0 2
TD 128
SFO1 125.77 MHz
FIDRES 205.052490 Hz
SW 208.688 ppm
FnMODE undefined
F2 - Processing parameters
SI 2048
SF 500.1300090 MHz
WDW SINE
SSB 0
LB 0.00 Hz
GB 0
PC 1.40
F1 - Processing parameters
SI 1024
MC2 QF
SF 125.7578240 MHz
WDW SINE
SSB 0
LB 0.00 Hz
GB 0
ppm
ppm

e

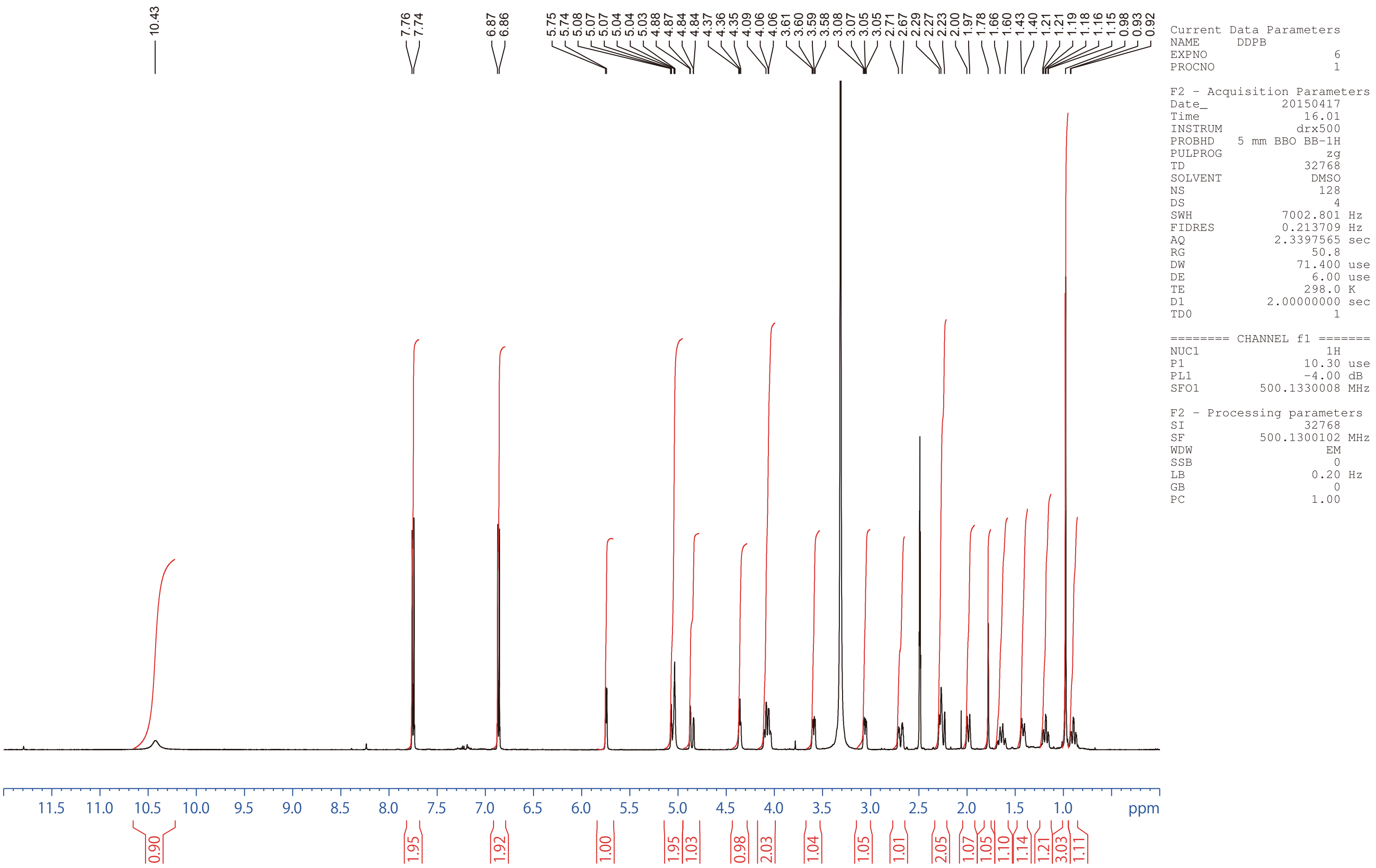

10.43
7.76 7.74
6.87 6.86
5.75 5.74 5.08 5.07 5.07 5.04 5.04 5.03 4.88 4.87 4.84 4.84 4.37 4.36 4.35 4.09 4.06 4.06 3.61 3.60 3.59 3.58 3.08 3.07 3.05 3.05 2.71 2.67 2.29 2.27 2.23 2.00 1.97 1.78 1.66 1.60 1.43 1.40 1.21 1.21 1.19 1.18 1.16 1.15 0.98 0.93 0.92
Current Data Parameters
NAME DDPB
EXPNO 6
PROCNO 1
F2 - Acquisition Parameters
Date_ 20150417
Time 16.01
INSTRUM drx500
PROBHD 5 mm BBO BB-1H
PULPROG zg
TD 32768
SOLVENT DMSO
NS 128
DS 4
SWH 7002.801 Hz
FIDRES 0.213709 Hz
AQ 2.3397565 sec
RG 50.8
DW 71.400 use
DE 6.00 use
TE 298.0 K
D1 2.00000000 sec
TD0 1
======== CHANNEL f1 =======
NUC1 1H
P1 10.30 use
PL1 -4.00 dB
SFO1 500.1330008 MHz
F2 - Processing parameters
SI 32768
SF 500.1300102 MHz
WDW EM
SSB 0
LB 0.20 Hz
GB 0
PC 1.00
11.5 11.0 10.5 10.0 9.5 9.0 8.5 8.0 7.5 7.0 6.5 6.0 5.5 5.0 4.5 4.0 3.5 3.0 2.5 2.0 1.5 1.0 ppm
0.90 1.95 1.92 1.00 1.95 1.03 0.98 2.03 1.04 1.05 1.01 2.05 1.07 1.05 1.10 1.14 1.21 3.03 1.11

f

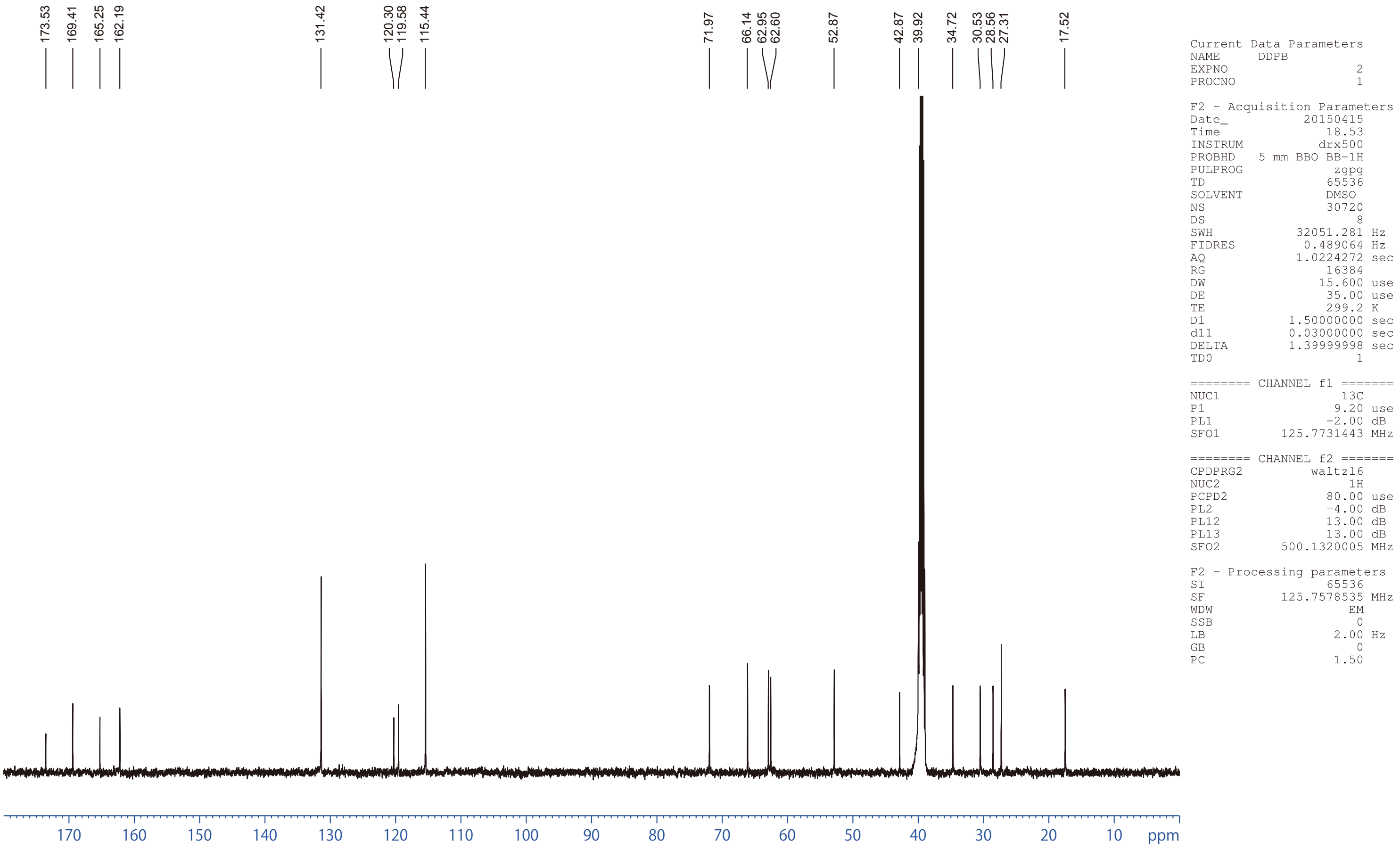

173.53 169.41 165.25 162.19
131.42
120.30 119.58 115.44
71.97
66.14 62.95 62.60
52.87
42.87 39.92
34.72 30.53 28.56 27.31
17.52
Current Data Parameters
NAME DDPB
EXPNO 2
PROCNO 1
F2 - Acquisition Parameters
Date_ 20150415
Time 18.53
INSTRUM drx500
PROBHD 5 mm BBO BB-1H
PULPROG zgpg
TD 65536
SOLVENT DMSO
NS 30720
DS 8
SWH 32051.281 Hz
FIDRES 0.489064 Hz
AQ 1.0224272 sec
RG 16384
DW 15.600 use
DE 35.00 use
TE 299.2 K
D1 1.50000000 sec
d11 0.03000000 sec
DELTA 1.39999998 sec
TD0 1
======== CHANNEL f1 =======
NUC1 13C
P1 9.20 use
PL1 -2.00 dB
SFO1 125.7731443 MHz
======== CHANNEL f2 =======
CPDPRG2 waltz16
NUC2 1H
PCPD2 80.00 use
PL2 -4.00 dB
PL12 13.00 dB
PL13 13.00 dB
SFO2 500.1320005 MHz
F2 - Processing parameters
SI 65536
SF 125.7578535 MHz
WDW EM
SSB 0
LB 2.00 Hz
GB 0
PC 1.50
170 160 150 140 130 120 110 100 90 80 70 60 50 40 30 20 10 ppm

g

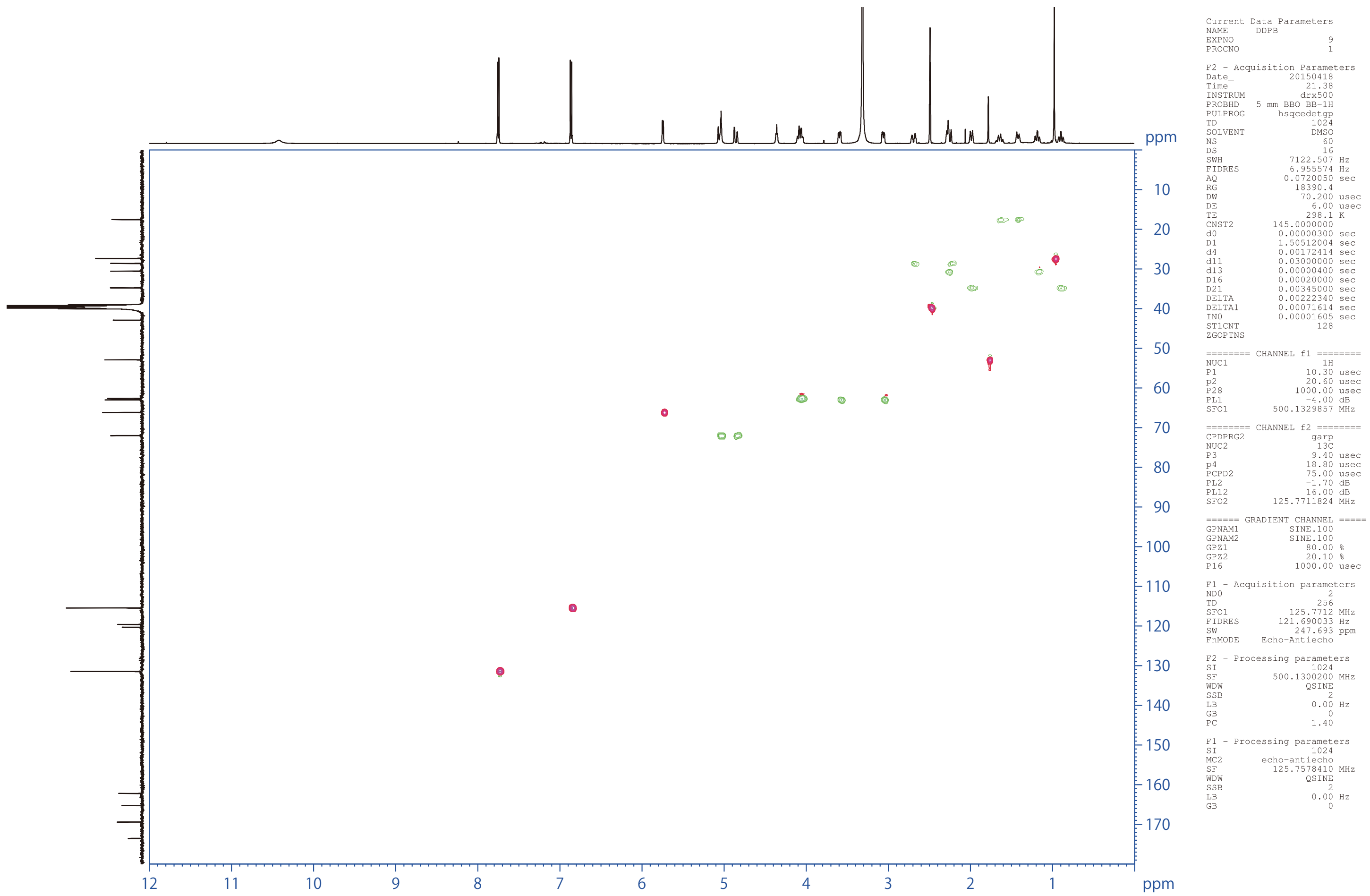
ppm
10
20
30
40
50
60
70
80
90
100
110
120
130
140
150
160
170
12
11
10
9
8
7
6
5
4
3
2
1
ppm
Current Data Parameters
NAME DDPB
EXPNO 9
PROCNO 1
F2 - Acquisition Parameters
Date_ 20150418
Time 21.38
INSTRUM drx500
PROBHD 5 mm BBO BB-1H
PULPROG hsqcedetgp
TD 1024
SOLVENT DMSO
NS 60
DS 16
SWH 7122.507 Hz
FIDRES 6.955574 Hz
AQ 0.0720050 sec
RG 18390.4
DW 70.200 usec
DE 6.00 usec
TE 298.1 K
CNST2 145.0000000
d0 0.00000300 sec
D1 1.50512004 sec
d4 0.00172414 sec
d11 0.03000000 sec
d13 0.00000400 sec
D16 0.00020000 sec
D21 0.00345000 sec
DELTA 0.00222340 sec
DELTA1 0.00071614 sec
IN0 0.00001605 sec
ST1CNT 128
ZGOPTNS
======== CHANNEL f1 ========
NUC1 1H
P1 10.30 usec
p2 20.60 usec
P28 1000.00 usec
PL1 -4.00 dB
SFO1 500.1329857 MHz
======== CHANNEL f2 ========
CPDPRG2 garp
NUC2 13C
P3 9.40 usec
p4 18.80 usec
PCPD2 75.00 usec
PL2 -1.70 dB
PL12 16.00 dB
SFO2 125.7711824 MHz
====== GRADIENT CHANNEL =====
GPNAM1 SINE.100
GPNAM2 SINE.100
GPZ1 80.00 %
GPZ2 20.10 %
P16 1000.00 usec
F1 - Acquisition parameters
ND0 2
TD 256
SFO1 125.7712 MHz
FIDRES 121.690033 Hz
SW 247.693 ppm
FnMODE Echo-Antiecho
F2 - Processing parameters
SI 1024
SF 500.1300200 MHz
WDW QSINE
SSB 2
LB 0.00 Hz
GB 0
PC 1.40
F1 - Processing parameters
SI 1024
MC2 echo-antiecho
SF 125.7578410 MHz
WDW QSINE
SSB 2
LB 0.00 Hz
GB 0

h

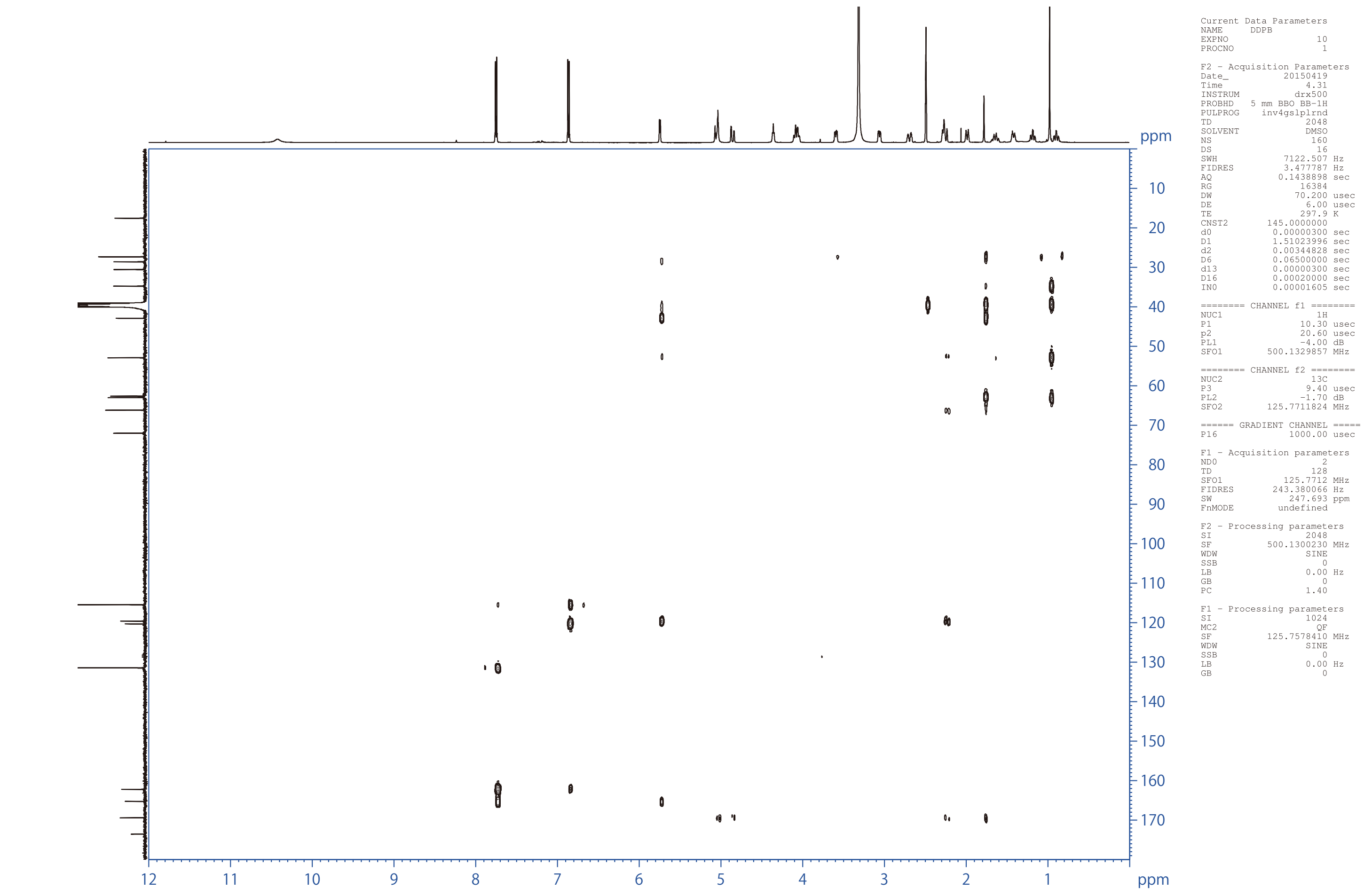
ppm
10
20
30
40
50
60
70
80
90
100
110
120
130
140
150
160
170
12
11
10
9
8
7
6
5
4
3
2
1
ppm
Current Data Parameters
NAME DDPB
EXPNO 10
PROCNO 1
F2 - Acquisition Parameters
Date_ 20150419
Time 4.31
INSTRUM drx500
PROBHD 5 mm BBO BB-1H
PULPROG inv4gslplrnd
TD 2048
SOLVENT DMSO
NS 160
DS 16
SWH 7122.507 Hz
FIDRES 3.477787 Hz
AQ 0.1438898 sec
RG 16384
DW 70.200 usec
DE 6.00 usec
TE 297.9 K
CNST2 145.0000000
d0 0.00000300 sec
D1 1.51023996 sec
d2 0.00344828 sec
D6 0.06500000 sec
d13 0.00000300 sec
D16 0.00020000 sec
IN0 0.00001605 sec
======== CHANNEL f1 ========
NUC1 1H
P1 10.30 usec
p2 20.60 usec
PL1 -4.00 dB
SFO1 500.1329857 MHz
======== CHANNEL f2 ========
NUC2 13C
P3 9.40 usec
PL2 -1.70 dB
SFO2 125.7711824 MHz
====== GRADIENT CHANNEL =====
P16 1000.00 usec
F1 - Acquisition parameters
ND0 2
TD 128
SFO1 125.7712 MHz
FIDRES 243.380066 Hz
SW 247.693 ppm
FnMODE undefined
F2 - Processing parameters
SI 2048
SF 500.1300230 MHz
WDW SINE
SSB 0
LB 0.00 Hz
GB 0
PC 1.40
F1 - Processing parameters
SI 1024
MC2 QF
SF 125.7578410 MHz
WDW SINE
SSB 0
LB 0.00 Hz
GB 0

i

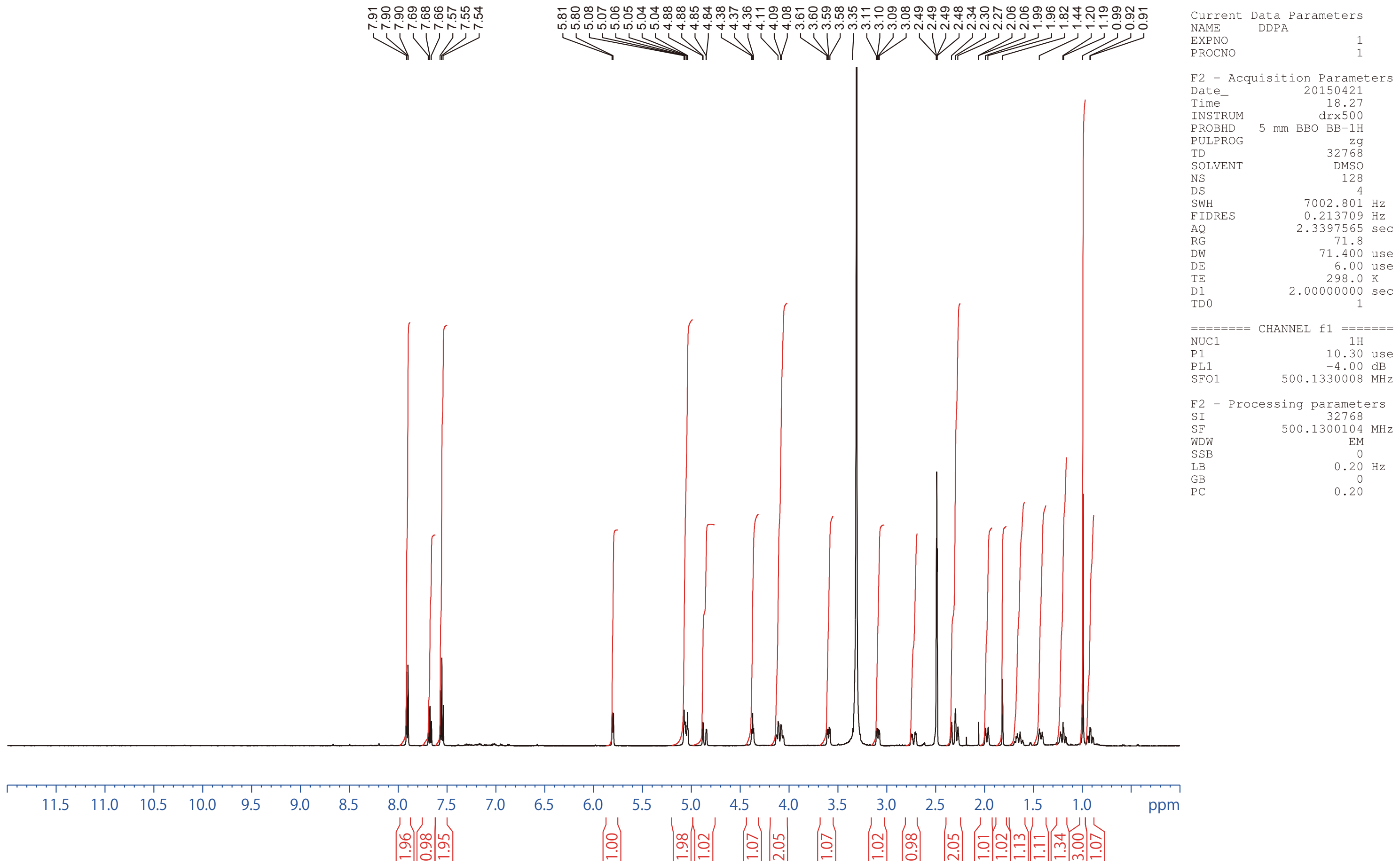

7.91
7.90
7.90
7.69
7.68
7.66
7.57
7.55
7.54
5.81
5.80
5.08
5.07
5.06
5.05
5.04
5.04
4.88
4.88
4.85
4.84
4.38
4.37
4.36
4.11
4.09
4.08
3.61
3.60
3.59
3.58
3.35
3.11
3.10
3.09
3.08
2.49
2.49
2.48
2.34
2.30
2.27
2.06
2.06
1.99
1.96
1.82
1.44
1.20
1.19
0.99
0.92
0.91
1.96
0.98
1.95
1.00
1.98
1.02
1.07
2.05
1.07
1.02
0.98
2.05
1.01
1.02
1.13
1.11
1.34
3.00
1.07
11.5 11.0 10.5 10.0 9.5 9.0 8.5 8.0 7.5 7.0 6.5 6.0 5.5 5.0 4.5 4.0 3.5 3.0 2.5 2.0 1.5 1.0 ppm
Current Data Parameters
NAME DDPA
EXPNO 1
PROCNO 1
F2 - Acquisition Parameters
Date_ 20150421
Time 18.27
INSTRUM drx500
PROBHD 5 mm BBO BB-1H
PULPROG zg
TD 32768
SOLVENT DMSO
NS 128
DS 4
SWH 7002.801 Hz
FIDRES 0.213709 Hz
AQ 2.3397565 sec
RG 71.8
DW 71.400 use
DE 6.00 use
TE 298.0 K
D1 2.00000000 sec
TD0 1
======== CHANNEL f1 =======
NUC1 1H
P1 10.30 use
PL1 -4.00 dB
SFO1 500.1330008 MHz
F2 - Processing parameters
SI 32768
SF 500.1300104 MHz
WDW EM
SSB 0
LB 0.20 Hz
GB 0
PC 0.20

j

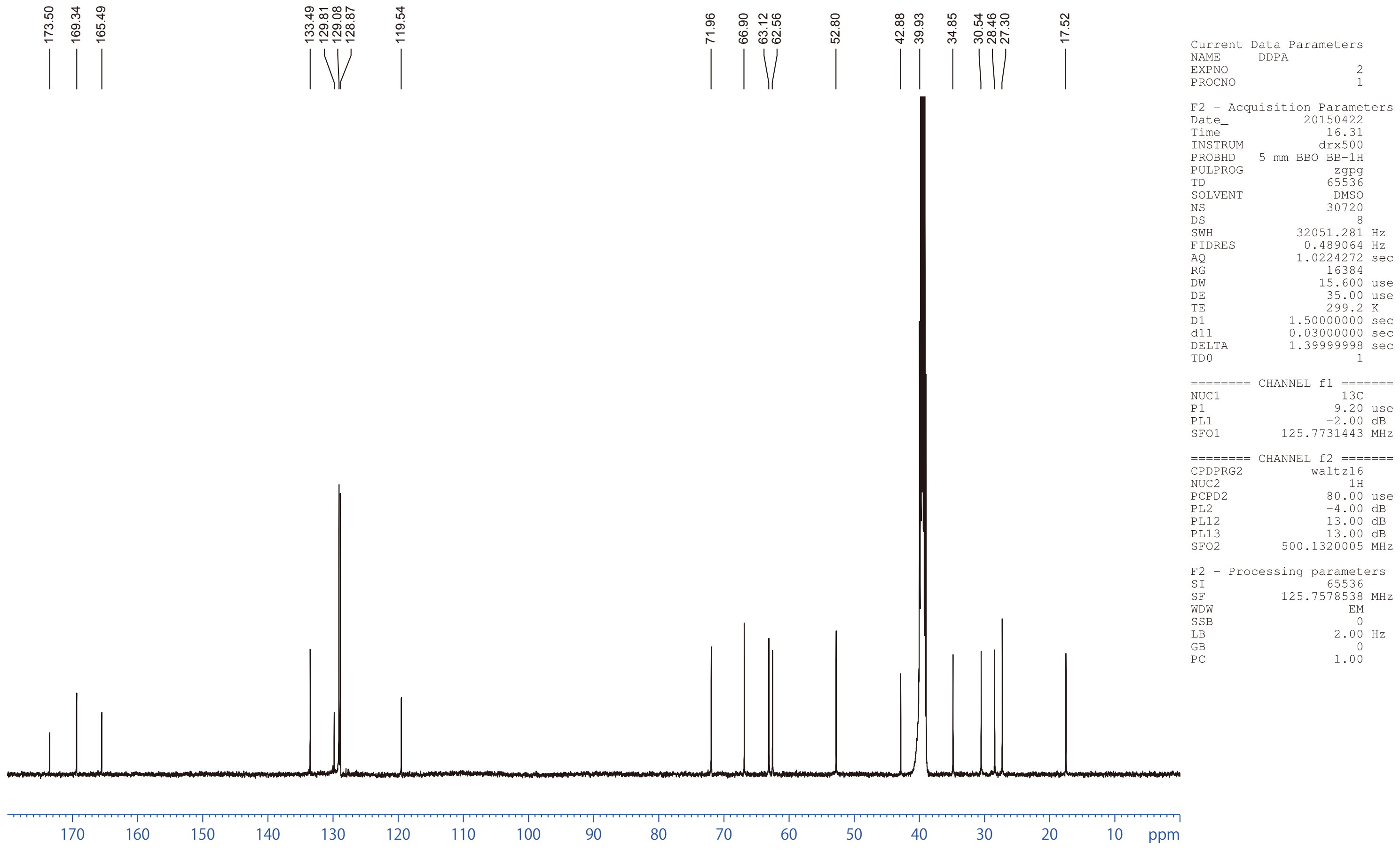

173.50
169.34
165.49
133.49
129.81
129.08
128.87
119.54
71.96
66.90
63.12
62.56
52.80
42.88
39.93
34.85
30.54
28.46
27.30
17.52
170 160 150 140 130 120 110 100 90 80 70 60 50 40 30 20 10 ppm
Current Data Parameters
NAME DDPA
EXPNO 2
PROCNO 1
F2 - Acquisition Parameters
Date_ 20150422
Time 16.31
INSTRUM drx500
PROBHD 5 mm BBO BB-1H
PULPROG zgpg
TD 65536
SOLVENT DMSO
NS 30720
DS 8
SWH 32051.281 Hz
FIDRES 0.489064 Hz
AQ 1.0224272 sec
RG 16384
DW 15.600 use
DE 35.00 use
TE 299.2 K
D1 1.50000000 sec
d11 0.03000000 sec
DELTA 1.39999998 sec
TD0 1
======== CHANNEL f1 =======
NUC1 13C
P1 9.20 use
PL1 -2.00 dB
SFO1 125.7731443 MHz
======== CHANNEL f2 =======
CPDPRG2 waltz16
NUC2 1H
PCPD2 80.00 use
PL2 -4.00 dB
PL12 13.00 dB
PL13 13.00 dB
SFO2 500.1320005 MHz
F2 - Processing parameters
SI 65536
SF 125.7578538 MHz
WDW EM
SSB 0
LB 2.00 Hz
GB 0
PC 1.00

k

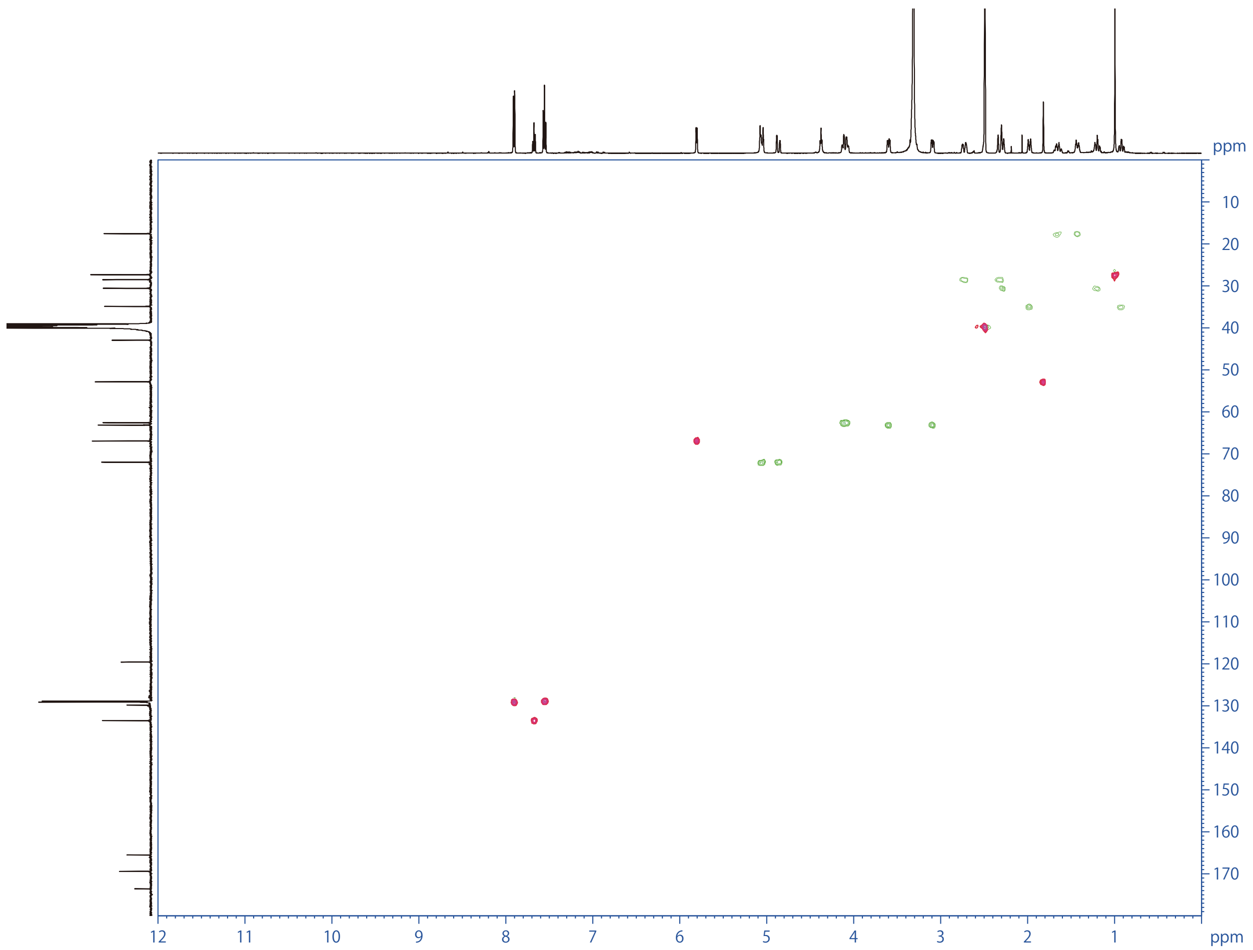


```
Current Data Parameters
NAME     DDPA
EXPNO                 8
PROCNO                1

F2 - Acquisition Parameters
Date_          20150429
Time              10.49
INSTRUM          drx500
PROBHD   5 mm BBO BB-1H
PULPROG      hsqcedetgp
TD                 1024
SOLVENT            DMSO
NS                   30
DS                   16
SWH            5787.037 Hz
FIDRES         5.651403 Hz
AQ            0.0886100 sec
RG              18390.4
DW               86.400 usec
DE                 6.00 usec
TE                298.0 K
CNST2       145.0000000
d0           0.00000300 sec
D1           1.48873603 sec
d4           0.00172414 sec
d11          0.03000000 sec
d13          0.00000400 sec
D16          0.00020000 sec
D21          0.00345000 sec
DELTA        0.00222340 sec
DELTA1       0.00071614 sec
IN0          0.00001800 sec
ST1CNT              128
ZGOPTNS

======== CHANNEL f1 ========
NUC1                 1H
P1                10.30 usec
p2                20.60 usec
P28             1000.00 usec
PL1               -4.00 dB
SFO1        500.1323377 MHz

======== CHANNEL f2 ========
CPDPRG2            garp
NUC2                13C
P3                 9.40 usec
p4                18.80 usec
PCPD2             75.00 usec
PL2               -1.70 dB
PL12              16.00 dB
SFO2        125.7699911 MHz

====== GRADIENT CHANNEL =====
GPNAM1         SINE.100
GPNAM2         SINE.100
GPZ1              80.00 %
GPZ2              20.10 %
P16             1000.00 usec

F1 - Acquisition parameters
ND0                   2
TD                  256
SFO1             125.77 MHz
FIDRES       108.506943 Hz
SW              220.862 ppm
FnMODE    Echo-Antiecho

F2 - Processing parameters
SI                 1024
SF          500.1300100 MHz
WDW               QSINE
SSB                   2
LB                 0.00 Hz
GB                    0
PC                 1.40

F1 - Processing parameters
SI                 1024
MC2       echo-antiecho
SF          125.7578390 MHz
WDW               QSINE
SSB                   2
LB                 0.00 Hz
GB                    0
```

l

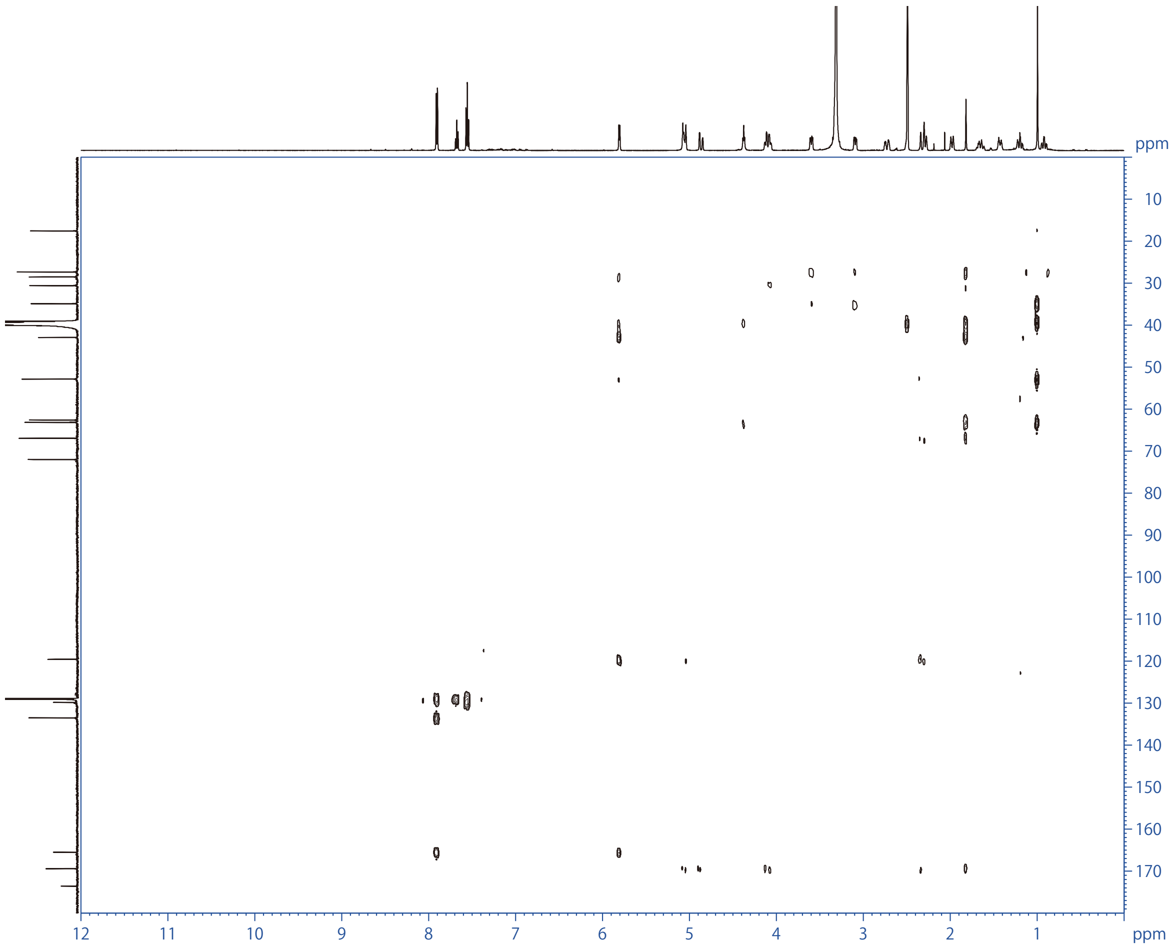


```
Current Data Parameters
NAME     DDPA
EXPNO                 9
PROCNO                1

F2 - Acquisition Parameters
Date_          20150429
Time              14.15
INSTRUM          drx500
PROBHD   5 mm BBO BB-1H
PULPROG     inv4gslplrnd
TD                 2048
SOLVENT            DMSO
NS                   60
DS                   16
SWH            5787.037 Hz
FIDRES         2.825702 Hz
AQ            0.1770836 sec
RG                16384
DW               86.400 usec
DE                 6.00 usec
TE                298.1 K
CNST2       145.0000000
d0           0.00000300 sec
D1           1.47747195 sec
d2           0.00344828 sec
D6           0.06500000 sec
d13          0.00000300 sec
D16          0.00020000 sec
IN0          0.00001800 sec

======== CHANNEL f1 ========
NUC1                 1H
P1                10.30 usec
p2                20.60 usec
PL1               -4.00 dB
SFO1        500.1323377 MHz

======== CHANNEL f2 ========
NUC2                13C
P3                 9.40 usec
PL2               -1.70 dB
SFO2        125.7699911 MHz

====== GRADIENT CHANNEL =====
P16             1000.00 usec

F1 - Acquisition parameters
ND0                   2
TD                  128
SFO1             125.77 MHz
FIDRES       217.013885 Hz
SW              220.862 ppm
FnMODE        undefined

F2 - Processing parameters
SI                 2048
SF          500.1300100 MHz
WDW                SINE
SSB                   0
LB                 0.00 Hz
GB                    0
PC                 1.40

F1 - Processing parameters
SI                 1024
MC2                  QF
SF          125.7578190 MHz
WDW                SINE
SSB                   0
LB                 0.00 Hz
GB                    0
```

Supplementary Figure 3. ^{1}H-NMR, ^{13}C-NMR, HSQC and HMBC spectra of 6, 7, and 8. The spectra were recorded for structure elucidation of **6**, **7**, and **8**. In all experiments DMSO-*d*6 was used as solvent and internal standard.

(a) ^{1}H-NMR spectrum of **6** (500 MHz).

(b) ^{13}C-NMR spectrum of **6** (125 MHz).

(c) HSQC spectrum of **6**.

(d) HMBC spectrum of **6**.

(e) ^{1}H-NMR spectrum of **7** (500 MHz).

(f) ^{13}C-NMR spectrum of **7** (125 MHz).

(g) HSQC spectrum of **7**.

(h) HMBC spectrum of **7**.

(i) ^{1}H-NMR spectrum of **8** (500 MHz).

(j) ^{13}C-NMR spectrum of **8** (125 MHz).

(k) HSQC spectrum of **8**.

(l) HMBC spectrum of **8**.

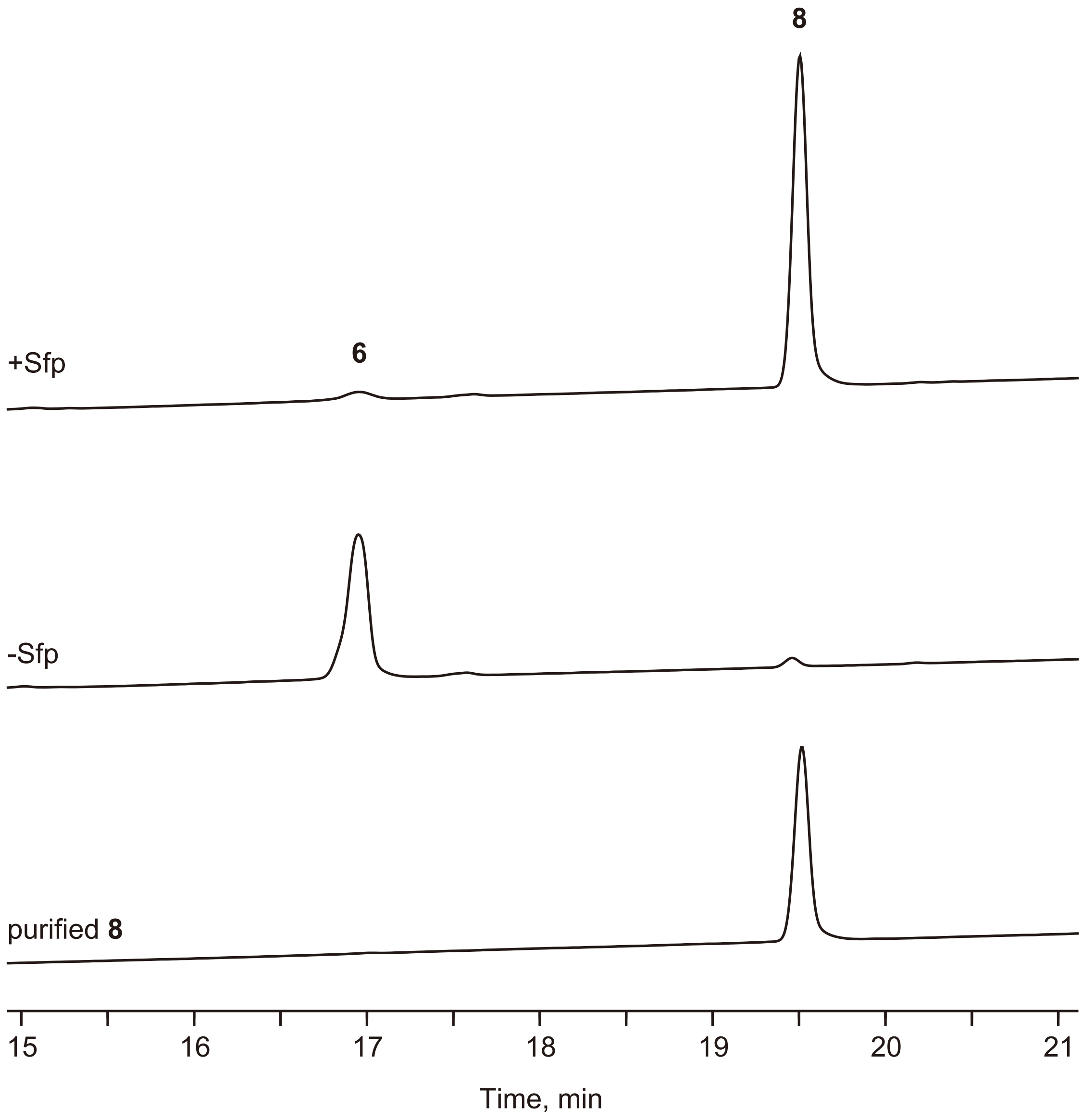

Supplementary Figure 4. *In vitro* assays for assessing the effects of Sfp on AstA activity. HPLC analysis (UV 220 nm) of the reaction products of AstA in the presence of BA and **6**, and purified **8**. Reactions were conducted in the presence or absence of Sfp.

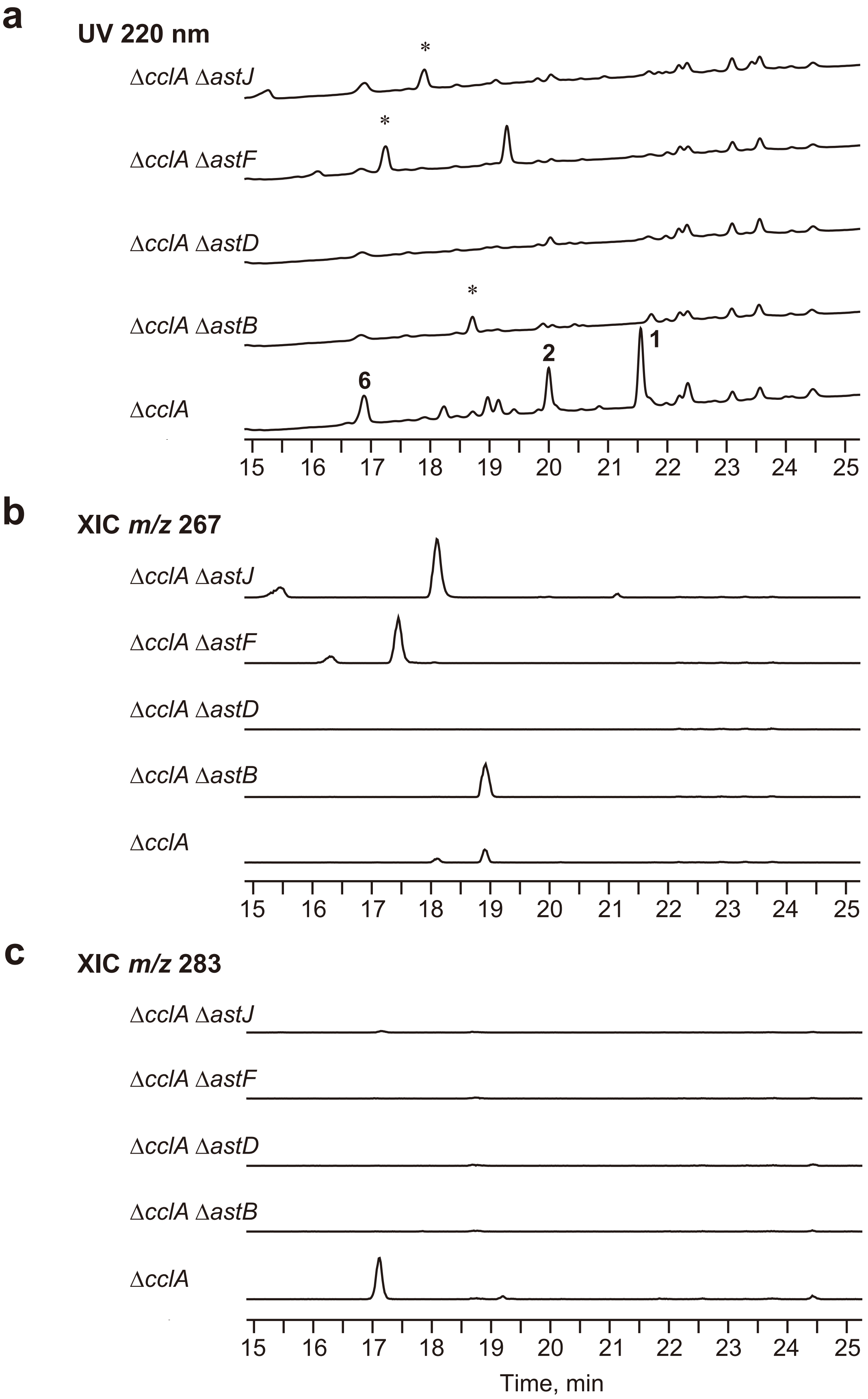

Supplementary Figure 5. Metabolic profile of culture extracts of the four strains that were disrupted for P450 (*astB, D, F,* and *J*) genes in the astellolide biosynthetic gene cluster. (**a**) HPLC profiles (UV 220 nm). (**b**) Extracted ion chromatograms (XIC) for *m/z* 267 (corresponding to dihydroxy confertifolin; $[M + H]^+$). (**c**) XIC for *m/z* 283 (**6**; $[M + H]^+$). The asterisk represents the accumulated compound corresponding to *m/z* 267 in the *astB*, *F*, and *J* gene disruption strains.

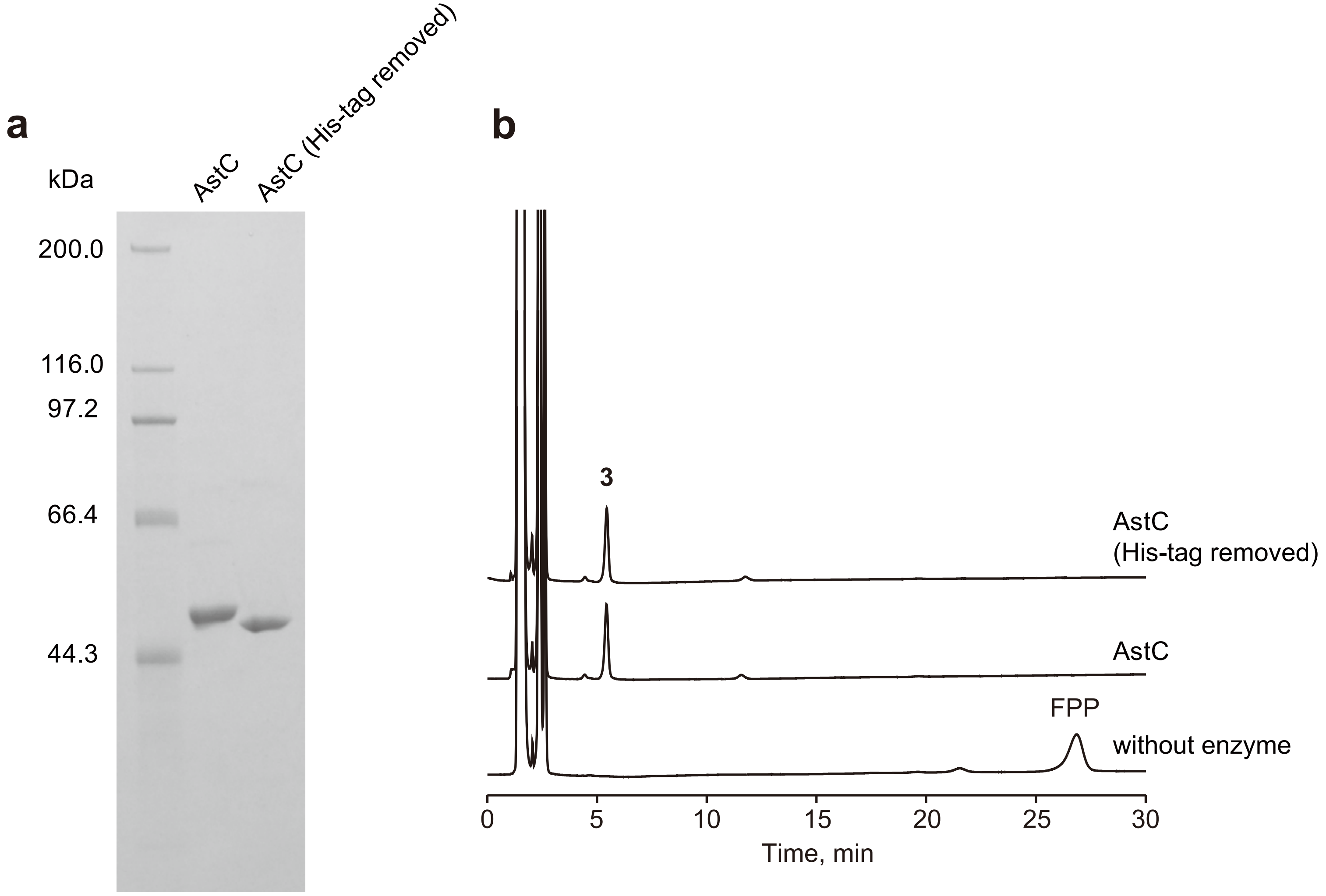

Supplementary Figure 6. HPLC analysis for assessing the effect of the His-tag on AstC activity. (**a**) SDS-PAGE analysis of AstC and AstC devoid of the His-tag. (**b**) HPLC analysis (UV 210 nm) of the reaction product of AstC or AstC devoid of the His-tag with FPP. As a negative control, enzymes were replaced by water.

Supplementary Table 1. ^{13}C and ^{1}H NMR chemical shifts of 3 (in D_2O).

	Drimanyl pyrophosphate (**3**)	
No.	^{13}C	^{1}H (J in Hz)
1	38.9	1.23, m
		1.92, brd (13.4)
2	21.3	1.52, m
		1.64, m
3	44.1	1.17, m
		1.41, m
4	35.4	-
5	54.3	1.11, d (12.6)
6	21.2	1.52, m
		1.71, m
7	36.2	2.08 - 2.11, m
8	138.9	-
9	140.1[a]	-
10	40.7	-
11	64.2[a]	4.40, brd (10.7)
		4.53, brd (10.7)
12	21.8	1.74, s
13	23.8	0.86, s
14	35.5	0.90, s
15	22.8	0.99, s

[a] $J_{C\text{-}P}$ couplings were observed.

Supplementary Table 2. ^{13}C and ^{1}H NMR chemical shifts of 6, 7, and 8 (in DMSO-*d*6).

	trihydroxy confertifolin (**6**)		dideacetyl astellolide B (**7**)		dideacetyl astellolide A (**8**)	
No.	^{13}C	^{1}H (*J* in Hz)	^{13}C	^{1}H (*J* in Hz)	^{13}C	^{1}H (*J* in Hz)
1	32.0	1.14, ddd (13.0, 13.0, 3.6)	30.5	1.18, ddd (13.0, 13.0, 3.3)	30.5	1.19, ddd (13.2, 13.2, 3.4)
		2.11, d (12.3)		2.28, m		2.29, d (13.2)
2	17.9	1.38, m	17.5	1.42, m	17.5	1.43, m
		1.58 - 1.62, m		1.63, m		1.65, m
3	37.6	1.08, ddd (14.2, 14.2, 4.2)	34.7	0.90, ddd (13.4, 13.4, 3.3)	34.9	0.92, ddd (13.4, 13.4, 3.5)
		1.58 - 1.62, m		1.99, d (13.4)		1.98, d (13.4)
4	38.8	-	39.9	-	39.9	-
5	54.5	1.48, s	52.9	1.78, brs	52.8	1.82, brs
6	61.9	4.36, d (4.8)	66.1	5.75, d (5.8)	66.9	5.81, d (5.8)
7	30.5	2.18, d (18.0)	28.6	2.25, d (18.2)	28.5	2.32, d (19.4)
		2.36, m		2.69, m		2.73, m
8	120.6	-	119.6	-	119.5	-
9	168.5	-	169.4	-	169.3	-
10	42.8	-	42.9	-	42.9	-
11	71.0	4.77, ddd (17.4, 3.5, 1.2)	72.0	4.86, dd (17.5, 1.9)	72.0	4.87, dd (17.6, 2.1)
		4.96, dt (17.4, 2.7)		5.05, m		5.05, m
12	174.0	-	173.5	-	173.5	-
13	28.0	0.99, s	27.3	0.97, s	27.3	0.99, s
14	66.1	3.26, d (11.4)	63.0	3.06, dd (10.6, 4.6)	63.1	3.09, dd (10.6, 4.8)
		3.81, d (11.4)		3.59, dd (10.6, 4.6)		3.60, dd (10.6, 4.8)
15	61.8	3.82, d (11.1)	62.6	4.07, m	62.6	4.10, m
		4.14, d (11.1)				
1'	-		165.3	-	165.5	-
2'	-		120.3	-	129.8	-
3',7'	-		131.4	7.75, d (8.8)	129.1	7.91, brd (7.3)
4',6'	-		115.4	6.86, d (8.8)	128.9	7.55, brt (7.7)
5'	-		162.2	-	133.5	7.68, brt (7.4)
6-OH		5.64		-		-
14-OH		5.40		4.36		4.37
15-OH		5.01		5.04		5.08
5'-OH		-		10.43		-

Supplementary Table 3. Strains used in this study.

Strain	Genotype	Source or reference
RkuptrP2-1 AF/P	Δ*ku70*::*ptrA* ΔAF *pyrG*$^{+}$	Ogawa et al., 2010
Δ*cclA*	Δ*ku70*::*ptrA* ΔAF Δ*cclA*::*pyrG*	Shinohara et al., 2016
Δ*cclA* Δ*pyrG*	Δ*ku70*::*ptrA* ΔAF Δ*cclA* Δ*pyrG*	This study
Δ*cclA* Δ*astA*	Δ*ku70*::*ptrA* ΔAF Δ*cclA* Δ*astA*::*pyrG*	This study
Δ*cclA* Δ*astB*	Δ*ku70*::*ptrA* ΔAF Δ*cclA* Δ*astB*::*pyrG*	This study
Δ*cclA* Δ*astC*	Δ*ku70*::*ptrA* ΔAF Δ*cclA* Δ*astC*::*pyrG*	This study
Δ*cclA* Δ*astD*	Δ*ku70*::*ptrA* ΔAF Δ*cclA* Δ*astD*::*pyrG*	This study
Δ*cclA* Δ*astE*	Δ*ku70*::*ptrA* ΔAF Δ*cclA* Δ*astE*::*pyrG*	This study
Δ*cclA* Δ*astF*	Δ*ku70*::*ptrA* ΔAF Δ*cclA* Δ*astF*::*pyrG*	This study
Δ*cclA* Δ*astG*	Δ*ku70*::*ptrA* ΔAF Δ*cclA* Δ*astG*::*pyrG*	This study
Δ*cclA* Δ*astH*	Δ*ku70*::*ptrA* ΔAF Δ*cclA* Δ*astH*::*pyrG*	This study
Δ*cclA* Δ*astI*	Δ*ku70*::*ptrA* ΔAF Δ*cclA* Δ*astI*::*pyrG*	This study
Δ*cclA* Δ*astJ*	Δ*ku70*::*ptrA* ΔAF Δ*cclA* Δ*astJ*::*pyrG*	This study
Δ*cclA* Δ*586*	Δ*ku70*::*ptrA* ΔAF Δ*cclA* Δ*586*::*pyrG*	This study
Δ*cclA* Δ*574*	Δ*ku70*::*ptrA* ΔAF Δ*cclA* Δ*574*::*pyrG*	This study

Supplementary Table 4. PCR primers used for the construction of gene disruption and *pyrG*-marker recycling cassettes.

Primer name	Sequence (5' to 3')[a]
cclA-LU	AAGGATGAGATCGGCTAGATTTGTG
cclA-LL	TCAGGGGAGCCTACATTGCCTTTGGACCTTGGACCGTCAGTTTG
cclA-RU	TGACGGTCCAAGGTCCAAAGGCAATGTAGGCTCCCCTGATGTAG
cclA-RL	TGAATTTGGCATGGGTTCTCATTC
cclA-LU-2	TCGCTGCTATATTTCCCAATGAAGTC
cclA-RL-2	CGGCTGCATACACTGGGACAGTTG
astA-LU	CACCAGTGGAGTGACAGATCAT
astA-LL	AGGGTACGTCTGTTGTAGGATACTGAAGATGAGGCCAG
astA-RU	CTTCTGAGGTGCAGTTGACTCCTATCGATCGTACTCTG
astA-RL	GGATCGATACTCACTACGGA
astA-LU-2	GACTCCCGACTCTGAATGATCT
astA-RL-2	CAGGATCGATACTCACTACGGA
astB-LU	CGAAAGGCAGTGTTTGATCGTCTGC
astB-LL	GTACGTCTGTTGTCTTCCCTTGGAAAAGGCTGACGGT
astB-RU	CTGAGGTGCAGTTCGCTCAAGCACACCAGTGGAGT
astB-RL	TGCCACATGCGTTTATGCGGAGG
astB-LU-2	AAAGTCGCCAGGAGCTTCGTCA
astB-RL-2	TGATCGAGCAGAGGATCGATGTCCT
astC-LU	TGCATACGGCAAGTAATTCCGCCA
astC-LL	GTACGTCTGTTGTTGGGAAGTGACTTGAGCATGGCAT
astC-RU	CTGAGGTGCAGTTTTCCGCCATGTCCTGGAGAGCA
astC-RL	AGCTGATCGGGTCTACTGGTACACC
astC-LU-2	ATCTGGCCCTTATCAGTTGGCTGCA
astC-RL-2	CACTTGACGTACCCAGGCCAAAGT
astD-LU	CCTGCAATCGGTAACAAGTACGGCT
astD-LL	GTACGTCTGTTGTGTGGTCCAGTACAAACTGCACCCA
astD-RU	CTGAGGTGCAGTTTCACACAGCACCTGTTGGATGCA
astD-RL	TCTCAAAGCGTACTGCAAGTTGGCA
astD-LU-2	TGGACGGATGATATGGAAGCCTTGC
astD-RL-2	AGCTTCGGCTTTTGTCCAGCAGA

astE-LU	TGGTCGAGTAAACCATGTACGCCGT
astE-LL	<u>GTACGTCTGTTGT</u>TGACCATGGCCTGGACGCTTTC
astE-RU	<u>CTGAGGTGCAGTT</u>AACCTTGTGGGCGTCGCATTCT
astE-RL	CCGGTCAGCAATGATCGTAGGCTC
astE-LU-2	GGCTGGAGTGATCGCCTCGATC
astE-RL-2	TCCGAGACCAACTTTTGCGCTCTG
astF-LU	ACCCCTTGCAAGAAGGTGGCTT
astF-LL	<u>GTACGTCTGTTGT</u>CGTTGCGTTGGTAAGTGTCTGGGT
astF-RU	<u>CTGAGGTGCAGTT</u>TGATCGGCGTGCTTTTTGAGGCAG
astF-RL	ACAGAGCGCAAAAGTTGGTCTCGGA
astF-LU-2	GTTGACAGCGGAGATGCTCCCTAC
astF-RL-2	TCGCGTTCCTGAAAGAGGCGTTCT
astG-LU	CAATCAGCCCATGAAGCCACACC
astG-LL	<u>GTACGTCTGTTGT</u>CGATCGATGGTGAAGCGTCCTCA
astG-RU	<u>CTGAGGTGCAGTT</u>CGCGCACTAAAGTCCCATGGCT
astG-RL	CGTTCATGCATACCGGAGTTCAGCT
astG-LU-2	CATGGATTGCGCTCTCAGGTAGGT
astG-RL-2	AGGAACCATCTCCAGGCACGTCA
astH-LU	AGCTGAACTCCGGTATGCATGAACG
astH-LL	<u>GTACGTCTGTTGT</u>AACAGTCAGCCCAGCCAAATCAC
astH-RU	<u>CTGAGGTGCAGTT</u>ACTGGAACGAAGGCCGCTTTTGA
astH-RL	ACGCGTTGTTGGAGTCCATTCGA
astH-LU-2	AGGCTGTGAACATCTGCCATCCAT
astH-RL-2	CTGCTAGCACCATCGGGAGGTT
astI-LU	TCGATGCGGCTAGAGTGCTCCA
astI-LL	<u>GTACGTCTGTTGT</u>TAAACGGGGGACTTCATGCTGGTG
astI-RU	<u>CTGAGGTGCAGTT</u>TCATCCGGCAGCTGAAGCAGAC
astI-RL	CATGGTCGGTTTGTGCCAAAGGTC
astI-LU-2	GAGGCCTTCTATGTTGCTGTGGCT
astI-RL-2	CAGAGATGGCTTGATCGTGACGCA
astJ-LU	AGATGCAGCCCTACCATCCCAAC
astJ-LL	<u>GTACGTCTGTTGT</u>AGCCTTCTGAACCAATCCACACAGG
astJ-RU	<u>CTGAGGTGCAGTT</u>GACACTGGTAAGGGCTGATTCCCCT

astJ-RL	GCTGCGTTCATCTCAATCTCGGGTG
astJ-LU-2	GCCATCGAACAAGTCCCACTCGTAG
astJ-RL-2	AAATGGCTCCCCAAATGCAAGGTC
586-LU	GAGGCAAACTTTTGCGTGGTCGAA
586-LL	GTACGTCTGTTGTCAATCTGTGCTAGCAGCGCGAGTT
586-RU	CTGAGGTGCAGTTCTCACTACGGACGGCCAGTCAGT
586-RL	TGGAACGTGCAGTTGATGGCGAAC
586-LU-2	GACCTCAACGCGGCCATTCAGG
586-RL-2	ACTTGGGATGACGCTTGCCTTCCA
574-LU	AACAGAGCCGTCACGCCATCTTC
574-LL	GTACGTCTGTTGTAGGATGCCGAGGACCCTTCACTG
574-RU	CTGAGGTGCAGTTTCACAGCTCGGAGCACCTTCC
574-RL	TGGAGGTCTTATGCGTTTGCTGGTC
574-LU-2	CACCCATCAGAACAGACAGCGCCTT
574-RL-2	GTGGTTGCTGACTCGCCGCTTAGA
pyrG-U	ACAACAGACGTACCCTGTGATGTTC
pyrG-L	AACTGCACCTCAGAAGAAAAGGATG

[a] Underlined sequences represent the additional nucleotides for fusion PCR

Supplementary Table 5. Primers used for qRT-PCR.

Primer name	Sequence (5' to 3')	target gene
astA qRT-F	CATCTGCGACTCGGGTGGTG	AO090026000585
astA qRT-R	TGGCCAGTACGGCTCACTGA	(*astA*)
astB qRT-F	TGGATGGTGGATTCGTTCCCGA	AO090026000584
astB qRT-R	CTGCGCATGAATCTGCTGACCGA	(*astB*)
astC qRT-F	CTTCGTAGCCATGCGAAGTCC	AO090026000582
astC qRT-R	CGACAAGATCGATGTCACCCGT	(*astC*)
astD qRT-F	ATACCAGGCAAGACCGTTGTCA	AO090026000581
astD qRT-R	TAGCGTCCTCGTCGAAACGTGG	(*astD*)
astE qRT-F	AGCGAAAGCGTCCAGGCCAT	AO090026000580
astE qRT-R	GCCAGTGATCCTCGTGGAGT	(*astE*)
astF qRT-F	CTACTGGTCGGGGCACATGGAC	AO090026000579
astF qRT-R	GCGCTAATCGACGTCCTGCACA	(*astF*)
astG qRT-F	GCCTGGAAGTCTTTGACGTGCCT	AO090026000578
astG qRT-R	GCGCAGACGACCCAACACCT	(*astG*)
astH qRT-F	CCATCGTAGCAGTGCAAGGCTC	AO090026000577
astH qRT-R	ACGTTCTGGCCGACGGACAT	(*astH*)
astI qRT-F	TCATCCGGCAGCTGAAGCAG	AO090026000576
astI qRT-R	CCGAGGCAAAGACCTCGTCGA	(*astI*)
astJ qRT-F	CTTGCATTGGAAGAAACCTCGCA	AO090026000575
astJ qRT-R	TGCCCAGTCTGCTGAACGCT	(*astJ*)
586 qRT-F	ATGGCGTCAATACGGGCTTC	AO090026000586
586 qRT-R	ATTGCCGGACGCGGTATCCT	
574 qRT-F	GTGAAGGGTCCTCGGCATCCT	AO090026000574
574 qRT-R	AGGAGTAGCCGTAGGTCTGGGA	
H2B qRT-F	AAGAAGCGTGGAAAGACCAGG	Histone 2B
H2B qRT-R	GACATGGCACGAGTGGAGATT	

Supplementary References

1. Ogawa, M., Tokuoka, M., Jin, F. J., Takahashi, T. & Koyama, Y. Genetic analysis of conidiation regulatory pathways in koji-mold *Aspergillus oryzae*. *Fungal Genet. Biol.* ***47***, 10–18 (2010).
2. Shinohara, Y., Kawatani, M., Futamura, Y., Osada, H. & Koyama, Y. An overproduction of astellolides induced by genetic disruption of chromatin-remodeling factors in *Aspergillus oryzae*. *J. Antibiot.* ***69***, 4-8 (2016).
